# Elucidation of a protein-protein interaction network involved in *Corynebacterium glutamicum* cell wall biosynthesis as determined by bacterial two-hybrid analysis

**DOI:** 10.1007/s10719-014-9549-3

**Published:** 2014-08-13

**Authors:** Monika Jankute, Charlotte V. Byng, Luke J. Alderwick, Gurdyal S. Besra

**Affiliations:** School of Biosciences, University of Birmingham, Edgbaston, Birmingham, B15 2TT UK

**Keywords:** Arabinogalactan, Biosynthesis, Cell wall, Corynebacteria, Mycobacteria, Protein-protein interactions

## Abstract

*Mycobacterium* species have a highly complex and unique cell wall that consists of a large macromolecular structure termed the mycolyl-arabinogalactan-peptidoglycan (mAGP) complex. This complex is essential for growth, survival and virulence of the human pathogen *Mycobacterium tuberculosis*, and is the target of several anti-tubercular drugs. The closely related species *Corynebacterium glutamicum* has proven useful in the study of orthologous *M. tuberculosis* genes and proteins involved in mAGP synthesis. This study examines the construction of a protein-protein interaction network for the major cell wall component arabinogalactan in *C. glutamicum* based on the use of a bacterial two-hybrid system. We have identified twenty-four putative homotypic and heterotypic protein interactions *in vivo*. Our results demonstrate an association between glycosyltransferases, GlfT1 and AftB, and interaction between the sub-units of decaprenylphosphoribose epimerase, DprE1 and DprE2. These analyses have also shown that AftB interacts with AftA, which catalyzes the addition of the first three arabinose units onto the galactan chain. Both AftA and AftB associate with other arabinofuranosyltransferases, including Emb and AftC, that elongate and branch the arabinan domain. Moreover, a number of proteins involved in arabinogalactan biosynthesis were shown to form dimers or multimers. These findings provide a useful recourse for understanding the biosynthesis and function of the mycobacterial cell wall, as well as providing new therapeutic targets.

**Electronic supplementary material:**

The online version of this article (doi:10.1007/s10719-014-9549-3) contains supplementary material, which is available to authorized users.

## Introduction

### Mycobacterium tuberculosis

the causative agent of tuberculosis (TB), remains a major cause of mortality and morbidity from a single infectious organism. In 2012, approximately 8.6 million people developed TB and 1.3 million died from the disease [1]. Emergence of multidrug-resistant [2], extensively drug resistant [2] and recently reported totally drug-resistant [3–5] clinical isolates has prompted the need for new drugs and drug targets. *M. tuberculosis* and other bacteria in the suborder of *Corynebacterineae* are characterized by a highly complex cell envelope. This cell wall is comprised of a cross-linked peptidoglycan (PG), covalently linked to arabinogalactan (AG) chains, which are further esterified by mycolic acids [6–8]. This macromolecular structure is often referred to as the mycolyl-arabinogalactan-peptidoglycan (mAGP) complex [9].

AG is composed predominantly of arabinofuranosyl (Ara*f*) and galactofuranosyl (Gal*f*) residues [10] and is covalently attached to PG *via* a specialized linker unit, L-Rha*p*-(1 → 4)-α-D-GlcNAc [7]. The galactan domain of AG is composed of approximately 30 alternating β (1 → 5) and β (1 → 6) Gal*f* residues connected in a linear fashion [11]. Three similar D-arabinan chains comprising roughly 30 Ara*f* residues each are attached to the galactan chain [12]. Since the AG structure is essential to *M. tuberculosis*, many gene deletion studies investigating AG have been performed in the closely related *Corynebacterium* genus, where aspects of AG biosynthesis are non-essential. Deletion studies in *C. glutamicum* demonstrated that the arabinan chains of AG are attached distinctively to the 8th, 10th, and 12th residue of the linear galactan chain [12]. Unlike most bacterial polysaccharides, AG lacks repeating units and is composed of a few distinct structural motifs, notably the terminal Ara_6_ motif, with the 5-OH of the t-Ara*f* and 2-Ara*f* residues representing sites for mycolylation [11, 8]. Collectively, AG, PG, and mycolic acids with additional outer layer lipids result in an exceptionally robust and hydrophobic cell wall structure. Importantly, a number of anti-TB drugs, such as ethambutol [13–15] and isoniazid [16, 17], target the biosynthesis of the mAGP complex.

The biosynthesis of AG involves the formation of the linkage unit synthesized on a decaprenyl phosphate lipid carrier (DP). Firstly, WecA transfers GlcNAc-1-P from the substrate UDP-GlcNAc-1-P onto the DP carrier [18, 19]. The rhamnosyltransferase WbbL then attaches the rhamnosyl residue to the DP-P-P-GlcNAc forming the full linker unit of AG, DP-P-P-GlcNAc-Rha [18, 20]. The linker unit serves as an acceptor for the sequential addition of roughly 30 Gal*f* residues. Bifunctional galactofuranosyltransferase (Gal*f*T) GlfT1 recognizes the linkage unit and transfers two Gal*f* residues to DP-P-P-GlcNAc-Rha yielding DP-P-P-GlcNAc-Rha-Gal*f*_2_ [21]. GlfT2 then attaches further Gal*f* residues acting both as a UDP-Gal*f*: β-D-(1 → 5) Gal*f*T and a UDP-Gal*f*:β-D-(1 → 6) Gal*f*T [22–24]. Arabinan biosynthesis employs decaprenylphosphoryl-D-arabinofuranose (DPA), the only known donor of Ara*f* residues in AG biosynthesis. The assembly of DPA has been recently investigated in detail [25, 26]. DPA biogenesis begins with UbiA transferring 5-phosphoribosyl-1-pyrophosphate to a DP forming decaprenylphosphoryl-5-phosphoribose (DPPR) [26]. DPPR is then dephosphorylated to decaprenyl-5-phosphoribose (DPR) by a putative phospholipid phosphatase [27]. DprE1 and DprE2 then catalyze the epimerization of DPR to DPA, consequently forming the essential sugar donor DPA [28–30]. A specialized arabinofuranosyltransferase (Ara*f*T) AftA transfers the first Ara*f* residue from the substrate molecule DPA onto the 8th, 10th and 12th Gal*f* residues of the galactan chain [12]. Further α (1 → 5)-linked Ara*f* residues are added by EmbA and EmbB in *M. tuberculosis* [31] or Emb in *C. glutamicum* [26]. Branching α (1 → 3) Ara*f*Ts, AftC and AftD, are responsible for α (1 → 3)-linked Ara*f* residues of the arabinan domain [32–35]. Finally, the terminal β (1 → 2) Ara*f* residues are transferred from DPA onto the arabinan domain by AftB [36, 37].

The structure and biogenesis of AG has been fairly well described, however, certain aspects of its biosynthesis remain poorly understood. For instance, the characterization of multi-protein complexes has been extremely limited, perhaps due to a number of cell wall biosynthesis proteins being transmembrane or membrane bound. In this study, we attempted to investigate the associations between *C. glutamicum* proteins involved in the assembly of AG by using the bacterial adenylate cyclase two-hybrid (BACTH) system [38]. This system is based on the functional complementation between two fragments of the adenylate cyclase to restore a cAMP signaling cascade in *Escherichia coli*. Importantly, BACTH is able to detect physical interactions between both cytoplasmic as well as membrane proteins [39–43].

Our data supports interactions between various proteins involved in AG biosynthesis. Moreover, we demonstrate a number of novel interactions between these proteins. Altogether, these results suggest that proteins involved in AG assembly are associated to one another through multiple interactions.

## Materials and methods

### Bacterial strains and growth conditions

All cloning steps were performed in *E. coli* XL-1 Blue cells (Invitrogen). The *E. coli cya* strain BTH101 ((F^−^, *cya*-99, *ara*D139, *gal*E15, *gal*K16, *rps*L1 (Str^r^), *hsd*R2, *mcr*A1, *mcr*B1) was used for the bacterial two-hybrid screen (Euromedex). *E. coli* strains were grown in Luria-Bertani (LB) medium at 30 °C or 37 °C as specified in the text. Plasmids were maintained with ampicillin (100 μg/ml) or kanamycin (50 μg/ml). LB agar reporter plates contained streptomycin (100 μg/ml), ampicillin (100 μg/ml), kanamycin (50 μg/ml), 5-bromo-4-chloro-3-indolyl-β-D-galactopyranoside (X-gal; 40 μg/ml) and isopropyl β-D-1-thiogalactopyranoside (IPTG; 0.5 mM). MacConkey plates (Difco^TM^) contained streptomycin (100 μg/ml), ampicillin (100 μg/ml), kanamycin (50 μg/ml), IPTG (0.5 mM) and maltose (1 %). M63 minimal media plates [44] were supplemented with streptomycin (50 μg/ml), ampicillin (50 μg/ml), kanamycin (25 μg/ml), X-gal (40 μg/ml), IPTG (0.5 mM) and maltose (0.2 %).

### Plasmid construction

All recombinant DNA methods were performed using standard protocols. Briefly, the genes involved in *C. glutamicum* AG biosynthesis were amplified from genomic DNA of *C. glutamicum* ATCC 13032. The plasmids have been constructed by inserting gene sequences of interest in pKT25 (T25 fusion at N-terminus), pKNT25 (T25 fusion at C-terminus), pUT18 (T18 fusion at C-terminus) and pUT18c (T18 fusion at N-terminus) [38], using oligonucleotides provided in Supplementary Table S1. The bacterial BACTH system kit was obtained from Euromedex and contained empty vectors together with positive control plasmids pKT25-*zip* and pUT18c-*zip*.

### Bacterial two-hybrid system

Two plasmids expressing recombinant proteins bearing N- or C- terminal T25 and T18 fusions were co-transformed into *E. coli* BTH101 cells (Table S2). Cells were spread on LB plates containing streptomycin (100 μg/ml), ampicillin (100 μg/ml), kanamycin (50 μg/ml) and incubated at 30 °C for 48 h. Several colonies were picked and used to inoculate 3 ml of LB supplemented with appropriate antibiotics and 0.5 mM IPTG. Cultures were grown overnight at 30 °C with shaking. Overnight cultures were washed three times in minimal media and spotted (2 μl) onto LB, MacConkey or M63 minimal media agar plates supplemented with appropriate antibiotics and nutrients. The β-galactosidase assay was performed as described elsewhere [44]. The values presented are the mean of 3 independent activity assays.

### Statistical analysis

The results are expressed as the means ± S.D. and were analyzed using a Student’s *t*-test to determine significant differences (*p* < 0.01) between samples.

## Results and discussion

### Network analysis of AG biosynthetic proteins

**Fig. 1:**
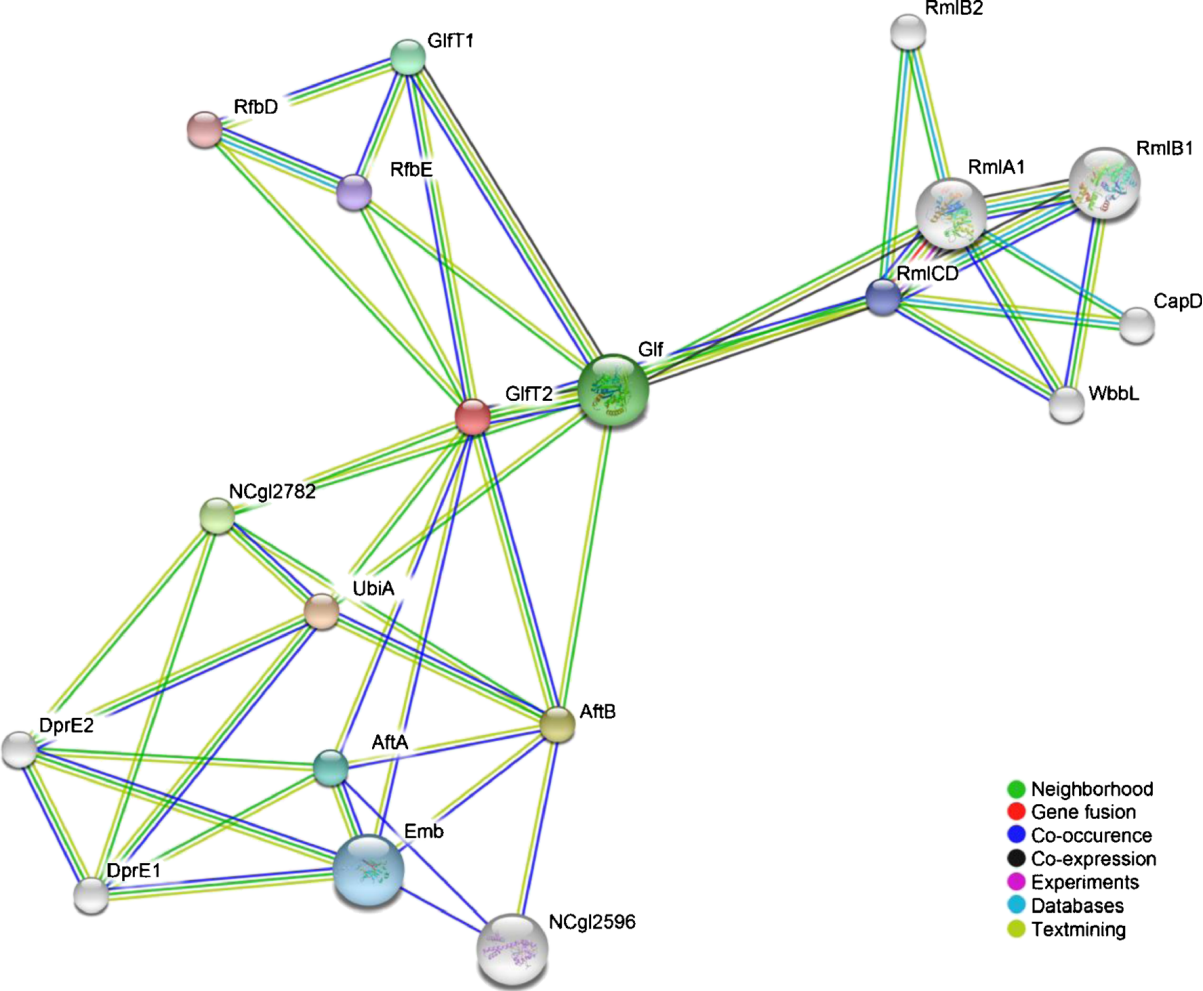
Network of *C. glutamicum* proteins found to be important for cell wall assembly round the GlfT2 protein as determined by STRING analysis. Lines connecting the nodes indicate various interaction data supporting the network, colored by evidence type

We initially aimed to identify whether any of the proteins involved in cell wall assembly have been predicted or demonstrated to make a functional network. Focusing on the list of proteins associated with AG biosynthesis we used the STRING database of interactions [45] to reveal a putative protein association network, with GlfT2 chosen as the network node (Fig. 1). The interaction patterns of proteins had a high confidence score (>0.7) and served as a basis for selection of *C. glutamicum* proteins that were further analyzed using the *in vivo* BACTH system. The generated network contained ABC family transporters (RfbD and RfbE), GT-A type Gal*f*Ts GlfT1, and proteins involved in rhamnose sugar donor formation, all centered on GlfT2. Transmembrane Ara*f*T Emb showed a strong evidence for interaction with AftA and AftB, as well as the uncharacterized protein NCgl2596. The network also contained a putative phospholipid phosphatase NCgl2782 and proteins involved in DPA synthesis: DprE1, DprE2 and UbiA.

### Bacterial two-hybrid analysis of AG proteins

To characterize the physical interactions between components of the *C. glutamicum* cell wall biosynthetic machinery, the following full-length proteins WecA, WbbL, GlfT1, GlfT2, AftA, AftB, AftC, AftD, Emb, UbiA, DprE1, and DprE2, were tested systematically for pair-wise interactions using BACTH. The known or predicted function of proteins is shown in Table 1. Each protein was fused to the fragment of the catalytic domain of chimeric adenylate cyclase (T25 or T18) of *Bordetella pertussis* at either the C- or N-terminus (Table S2). Interaction between two hybrid proteins leads to reconstitution of the fragments of adenylate cyclase resulting in restoration of cAMP production in a *E. coli cya* mutant [38]. The resulting cAMP forms a complex with the catabolite activator protein and binds to various promoters, thus regulating transcription of several genes, including the lactose and maltose operons. The activation of these operons can be detected on selective agar plates or using a β-galactosidase assay. Importantly, this bacterial two-hybrid system was shown to be suitable to detect interactions between cytoplasmic, as well as transmembrane or membrane associated proteins [46, 47].

**Table 1:** Predicted topology and function of *C. glutamicum* proteins described in this study

Protein	Predicted topology	Function
WecA	transmembrane	UDP-GlcNAc-1-phosphatetransferase
WbbL	soluble	α-3-L-rhamnosyltransferase
GlfT1	soluble	UDP-galactofuranosyltransferase
GlfT2	soluble	UDP-galactofuranosyltransferase
AftA	transmembrane	arabinofuranosyltransferase
AftB	transmembrane	arabinofuranosyltransferase
AftC	transmembrane	arabinofuranosyltransferase
AftD	transmembrane	arabinofuranosyltransferase
Emb	transmembrane	arabinofuranosyltransferase
DprE1	soluble	decaprenylphosphoryl-α-D-ribose 2'-oxidase
DprE2	soluble	decaprenylphosphoryl-D-2-keto erythro pentose reductase
UbiA	transmembrane	decaprenyl-phosphate 5-phosphoribosyltransferase

Despite several attempts, we did not succeed in constructing pKT25 and pUT18 derivatives expressing UbiA and AftD proteins, respectively. This is probably due to the toxicity of hybrid proteins to bacterial cells when expressed at high levels, which is especially true of membrane proteins. Moreover, the UbiA-T18^N^ and UbiA-T18^C^ hybrid proteins, when co-expressed with several other hybrid proteins, appeared to reduce down bacterial cell growth suggesting that overproduction of UbiA is toxic to *E. coli* cells.

**Fig. 2:**
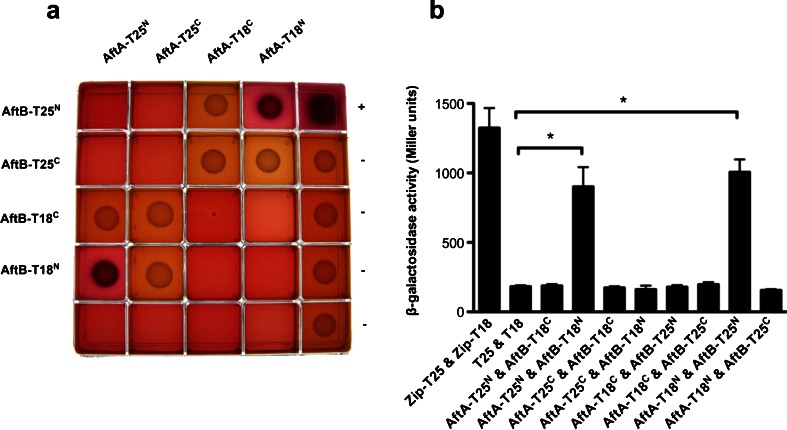
BACTH analysis of interactions between AftA and AftB proteins from *C. glutamicum*. The genes encoding full-length proteins were fused in frame with adenylate cyclase T25 or T18 fragments at N- or C-terminus and expressed in *E. coli cya* ^*−*^ BTH101. a Co-transformants containing two plasmids encoding putative interaction partners were spotted onto selective MacConkey agar supplemented with appropriate antibiotics, 0.5 mM IPTG and 1 % maltose. Plates were incubated at 30 °C for 48 h. Protein-protein interactions are indicated by red colonies through the reconstitution of adenylate cyclase catalytic domain. A strain co-expressing T25 and T18 fragments fused to leucine zipper domain was used as positive control (+), whereas empty pKT25-pUT18, pKT25-pUT18c, pKNT25-pUT18, and pKNT25-pUT18c were used as negative controls (−). b The efficiencies of functional complementation between hybrid proteins were quantified by measuring β-galactosidase activities in suspensions of toluene treated *E. coli* BTH101 harboring the corresponding plasmids. Results are expressed in Miller units and are the mean ± standard deviation of at least three independent experiments. Statistical significance was determined by Student’s *t*-test (p < 0.01)

To examine putative interactions between the hybrid proteins, *E. coli* BTH101 cells were co-transformed with pairs of recombinant plasmids (Table S2). In total, 577 pairs were screened for protein-protein interactions *in vivo*. All co-transformants, together with the positive and negative controls, containing either pKT25-*zip*/pUT18c-*zip* or empty pKT25/pUT18, were then spotted onto selective agar plates and the coloration of the colonies observed after 48 h of growth at 30 °C. In the absence of association between T25 and T18 fragments colonies appear white, whereas they are blue or red when functional complementation occurs. Representative plates from the screening are shown in Fig. 2 and Supplementary Fig. S1-S13. Efficiency of the functional complementation between T25 and T18 domains was quantified by measuring β-galactosidase activity (Fig. S1-S13). Ultimately, 50 pairs of hybrid proteins resulted in a positive signal representing 24 putative homotypic and heterotypic protein-protein interactions. The interaction results from the assays are summarized in Table 2.

**Table 2:** Protein-protein interactions between *C. glutamicum* AG biosynthetic proteins determined by BACTH

	WecA	WbbL	GlfT1	GlfT2	AftA	AftB	AftC	AftD	UbiA	DprE1	DprE2	Emb
WecA	✓											
WbbL	-	-										
GlfT1	-	-	✓									
GlfT2	-	-	-	✓								
AftA	-	-	-	-	✓							
AftB	✓	-	✓	-	✓	✓						
AftC	✓	-	-	-	✓	✓	✓					
AftD	-	-	-	-	-	-	-	-				
UbiA	✓	✓	-	-	✓	✓	✓	-	-			
DprE1	-	-	-	-	-	-	-	-	-	✓		
DprE2	-	-	✓	-	✓	✓	✓	-	-	✓	✓	
Emb	✓	-	-	-	✓	✓	-	-	-	-	-	-

The positive interaction is indicated as (✓), whereas the lack of interaction is marked as (−)

### Self-association of *C. glutamicum* cell wall biosynthesis proteins

Among all the tested proteins, dimerization or multimerization of WecA, GlfT1, GlfT2, AftA, AftB, AftC, DprE1, and DprE2 have been demonstrated employing BACTH. Co-expression of transmembrane WecA-T25^C^ and WecA-T18^C^ hybrid proteins restored a *cya* ^+^ phenotype and synthesis of cAMP in the *E. coli* cells, resulting in blue and red colonies on LB/M63-Xgal and MacConkey media, respectively (Fig. S1a-c). The β-galactosidase assay revealed a significant increase in β-galactosidase activity (487 ± 47 Miller units) when compared to the negative control (86 ± 11 Miller units), containing empty pKT25, pKNT25, pUT18, and pUT18c plasmids (Fig. S1d). Importantly, the transmembrane fusions have to be correctly inserted into the plasma membrane with the T18 and T25 domains facing the cytoplasm in order an interaction to be detected. Therefore, suggesting that the C- terminus of GlcNAc-1-phosphate transferase WecA is cytoplasmic. This is in agreement with the predicted topology of WecA [48]. Physical self-dimerization or multimerization *in vivo* was also demonstrated for GlfT1 and GlfT2. Consistently, GlfT1-T25^N^ and GlfT1-T18^C^, GlfT1-T25^N^ and GlfT1-T18^N^, GlfT1-T25^C^ and GlfT1-T18^N^, GlfT2-T25^N^ and GlfT2-T18^C^, and GlfT2-T25^C^ and GlfT2-T18^N^ hybrids restored *lac* ^+^ and *mal* ^+^ phenotypes and resulted in significant β-galactosidase activity ranging from 266 ± 69 to 679 ± 118 Miller units (Fig. S1e-h). Recently, the structure of the polymerizing GlfT2 orthologue in *M. tuberculosis* has been solved revealing its assembly as a homotetramer [23], thus supporting the results obtained in this BACTH study.

Transmembrane AftA, AftB and AftC, proteins also tested positive for self-association. Co-expression of AftA-T25^N^ and AftA-T18^N^, AftB-T25^N^ and AftB-T18^N^, AftC-T25^C^ and AftC-T18^C^ combinations yielded β-galactosidase activity of 714 ± 92, 1185 ± 265, and 398 ± 23 Miller units, respectively (Fig. S2). The C-terminal region of AftA and AftB is predicted to be directed towards the periplasm [37, 12], therefore the lack of interaction between fusion pairs carrying C-terminal T25 or T18 fragment was expected. In addition, BACTH experiments propose that the N- termini of AftA and AftB are cytoplasmic. In contrast to AftA and AftB, AftC is characterized by the absence of a periplasmic C- terminal extension [33]. Hence it is unsurprising that multimerization of AftC is observed with the fusion proteins tagged at the C- terminus. Interestingly, no evidence for homodimerization could be obtained for Emb and AftD. Finally, DprE1 and DprE2, both involved in DPA synthesis, appeared positive for self-interaction. DprE1-T25^N^ and DprE1-T18^C^, DprE1-T25^C^ and DprE1-T18^N^ fusions, as well as all four pairs of hybrid proteins co-expressing DprE2 led to a strong *lacZ* induction (ranged between 291 ± 33 and 1156 ± 54 Miller units), significantly exceeding the negative control (Fig. S3).

### *In vivo* interaction network among AG proteins

Next, we examined the interactions between different proteins involved in AG biosynthesis. Our results indicate that in addition to homodimerization, WecA is also able to interact with multiple partners of AG biosynthesis (Table 2). WecA-T18^C^ hybrid, when co-expressed with AftB-T25^N^, AftC-T25^C^, Emb-T25^N^, and UbiA-T25^C^ yielded significant β-galactosidase activities 1082 ± 268, 1047 ± 186, 1346 ± 217, and 1018 ± 137 Miller units, respectively (Fig. S4-5). BACTH also revealed an interaction with the rhamnosyltransferase WbbL, when UbiA hybrids were used as the bait. Co-transformation of WbbL-T18^C^ or WbbL-T18^N^ together with UbiA-T25^C^ led to a restoration of cAMP cascade with β-galactosidase activities of 1094 ± 93 and 1195 ± 78 Miller units, respectively (Fig. S6a-d). Our studies have demonstrated the physical interaction between GlfT1 and AftB (Fig. S6e-h), as well as the DprE2 involved in DPA formation (Fig. S7a-d). Recent studies reported the physical interaction between GlfT1 and Rv3789, a small multidrug resistance-like transporter [49]. Rv3789 was proposed to target and stabilize the membrane associated GlfT1 [49]. Further experiments demonstrated evidence for a physical interaction between UbiA and AftA-T25^N^ (387 ± 22 Miller units) (Fig. S8), AftB-T25^N^ (1015 ± 185 Miller units) (Fig. S10) and AftC- T25^C^ (755 ± 118 Miller untis) (Fig. S12), responsible for the biosynthesis of the arabinan domain of AG. Most of these Ara*f*Ts could also establish multiple interactions with each other. AftA, which primes the galactan chain of AG, associated with Emb, AftC and AftB (Fig. S7-9). In addition, AftB also interacted with Emb and AftC hybrid proteins (Fig. S10-11). Finally, *C. glutamicum* DprE1 was also found to strongly interact with DprE2. DprE1-DprE2 association has been identified with seven different plasmid combinations resulting in a significant β-galactosidase activity ranging between 637 ± 52 and 1027 ± 86 Miller units (Fig. S13). Previous studies reported that orthologues of DprE1 and DprE2 in *M. tuberculosis* were able to catalyze the epimerization reaction *in vitro*, however, neither protein alone was sufficient to support this activity [49]. Thus, strongly suggesting that DprE1 and DprE2 work in concert to catalyze the conversion of DPR to DPA. However, when the same *M. tuberculosis* orthologues were experimentally tested for interaction using BACTH, co-transformants yielded negative results [49].

## Conclusions

**Fig. 3:**
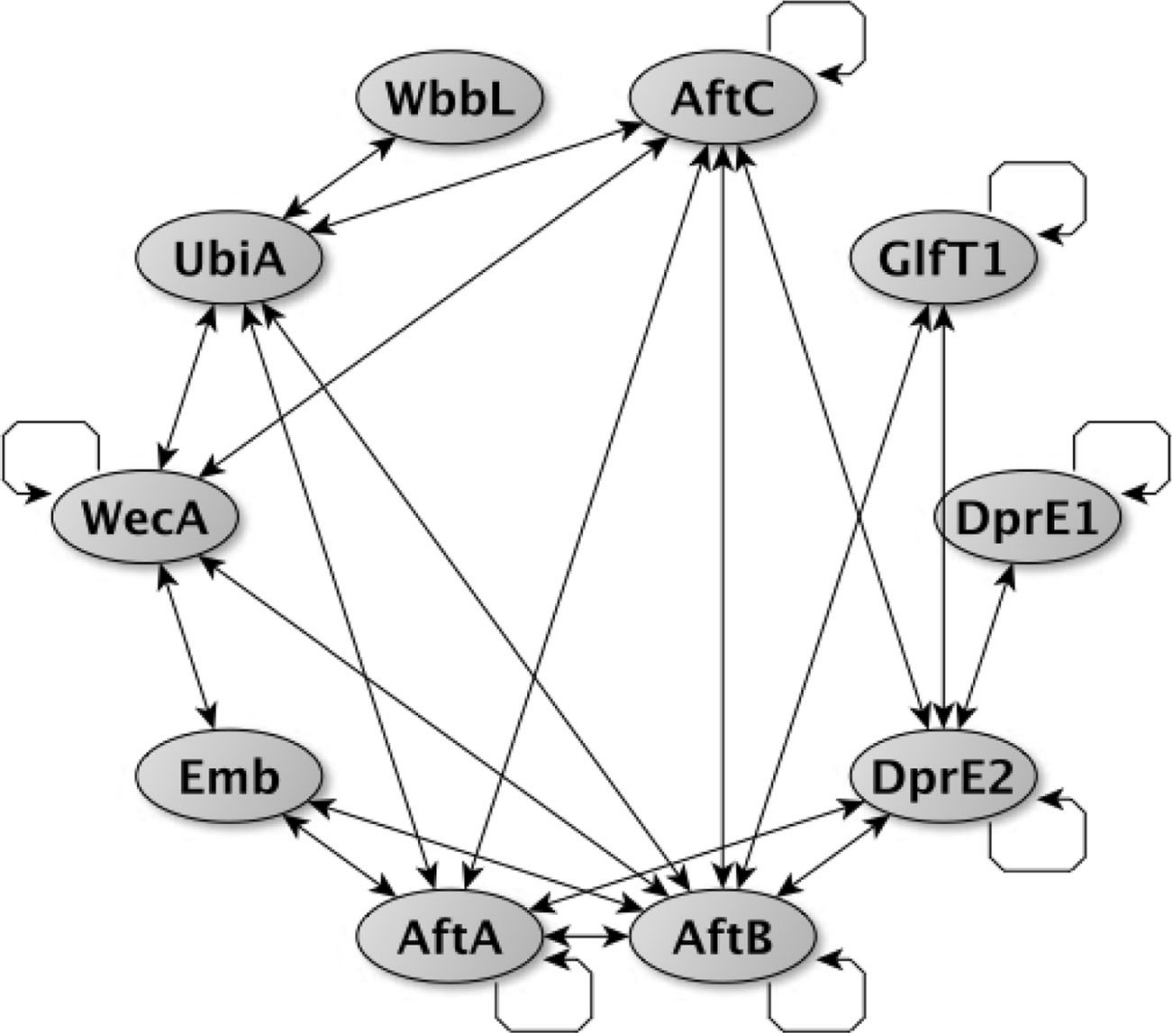
An interaction network of *C. glutamicum* proteins involved in AG biosynthesis generated using yEd graph editor software. The circular arrows indicate self-association

The majority of bacterial cell wall surface polysaccharides are built on a carrier lipid in the cytosolic side of the plasma membrane. Although it is not fully clear how and when these polymers are translocated to the periplasm, one could speculate that anchoring these macromolecules to the membrane positions them closely to the transporters and glycosyltransferases, therefore, promoting productive export across the plasma membrane. Formation of multi-protein complexes, that contain glycosyltransferases, enzymes forming its sugar nucleotides and transporters, is expected to be beneficial for the bacterial cell, since the tight arrangement of the biosynthetic reactions would retain productivity and accuracy of the polymerization process. We have demonstrated that the proteins responsible for the formation of the AG linker unit, WecA and WbbL, form a complex with decaprenylphosphoryl-5-phosphoribose synthase UbiA at the cytoplasmic membrane (Fig. 3). WecA and UbiA directly employ DP for the linker unit and DPPR formation, respectively [18, 30, 50]. Proximal interactions between WecA, WbbL and UbiA could perhaps facilitate synchronized utilization of DP for coordinated AG biosynthesis. In addition, UbiA show evidence for physical interaction between AftA, AftB and AftC proteins, which utilize DPA as a substrate (Fig. 3). It is possible that this multi-protein complex formation assists a mechanism similar to substrate channeling, where intermediary metabolic products of one enzyme are passed directly to another enzyme. Other DPA forming proteins, DprE1 and DprE2, showed evidence for a physical interaction. Interestingly, while both DprE1 and DprE2 are required for the epimerization reaction, there is evidence that *C. glutamicum* NCgl1429 may play a similar function to DprE2 [28]. Investigation into potential DprE1-NCgl1429 complexes could provide insight into this gene redundancy. Notably, GT-A glycosyltransferases GlfT1 and GlfT2 showed evidence for homodimerization using BACTH. GlfT1 transfers the first two Gal*f* residues to the linker unit, whereas GlfT2 is responsible for addition of approximately 30 Gal*f* residues in a linear chain. The recent crystal structure of *M. tuberculosis* GlfT2 in its apo-form and in complex with UDP, established its homotetrameric architecture [23]. Finally, AftA, AftB, AftC and Emb proteins involved in the assembly of arabinan domain in AG, indeed form a multi-protein complex at the inner membrane (Fig. 3). One could speculate that such a sophisticated complex would maintain the efficiency and fidelity of AG polymerization.

BACTH is a powerful technique for the investigation of protein-protein associations, however, several important notes should be highlighted regarding the significance of the interaction data obtained from BACTH. Firstly, the lack of *lacZ* induction might be a result of plasmid instability, insoluble or dissipating fusions, and not the lack of direct physical interaction. Therefore, the hybrid proteins that test negative for interactions may still interact *in vivo*. Moreover, since the output of the interaction – cAMP – requires to be generated in the cytoplasm, these negative results may also result from the incorrect topological orientation of functional T25 and T18 domains into plasma membrane. In addition, using BACTH, the fusion proteins are overexpressed when compared to the expression levels of native cells. Under these conditions, BACTH could have revealed a number of weak interactions between AG biosynthetic proteins. Although such associations would not take place at low protein concentrations, they can still occur when AG is being synthesized, where the local concentrations of proteins should be significantly higher. Finally, it is possible that some of the indentified interactions are a consequence of non-specific interactions initiated by endogenous *E. coli* host proteins that act as a tethering agent. These indirect associations, caused by a third protein, cannot be simply rejected.

In conclusion, our findings here suggest that enzymes involved in *C. glutamicum* cell wall assembly and precursor formation form complicated multi-protein complexes. We have identified 24 interactions *in vivo* between 12 proteins responsible for AG biosynthesis using BACTH. The challenge for the future will be to discover precisely how each of these multi-protein complexes form and function to synthesize and translocate AG.

## Electronic supplementary material

Below is the link to the electronic supplementary material.

ESM 1 (PDF 2372 kb)

## Abbreviations

AG: arabinogalactan
Ara*f*: arabinofuranosyl
Ara*f*T: arabinofuranosyltransferase
BACTH: bacterial adenylate cyclase two-hybrid
DP: decaprenyl phosphate
DPA: decaprenylphosphoryl-D-arabinofuranose
DPPR: decaprenylphosphoryl-5-phosphoribose
DPR: decaprenyl-5-phosphoribose
Gal*f*: galactofuranosyl
Gal*f*T: galactofuranosyltransferase
mAGP: mycolyl-arabinogalactan-peptidoglycan
PG: peptidoglycan
TB: tuberculosis
