# Supplementary material for: Elucidation of a protein-protein interaction network involved in Corynebacterium glutamicum cell wall biosynthesis as determined by bacterial two-hybrid analysis
Source: Glycoconj J. 2014 Aug 13;31(6):475–83. doi: 10.1007/s10719-014-9549-3 (PMC4213368; doi:10.1007/s10719-014-9549-3)
Supplement: Supplementary file 1 — (PDF 2372 kb) [file 10719_2014_9549_MOESM1_ESM.pdf]

SUPPLEMENTARY MATERIAL

Elucidation of a protein-protein interaction network involved in *Corynebacterium glutamicum* cell wall biosynthesis as determined by bacterial two-hybrid analysis

Monika Jankute, Charlotte V. Byng, Luke J. Alderwick, and Gurdyal S. Besra

Supplementary Tables and Figures

Table S1 Oligonucleotides used in this study

Plasmid[a]	Primer sequence (5' – 3')[b]	Origin
pKT25-*wecA*	5'-CATGCATGTCTAGAGATGGGAGTCGGTTTCGCG-3'<br>5'-CATGCATGGAATTCTTAATCAAGTTTGGCGGCT-3'	This study
pKNT25-*wecA*	5'-CATGCATGAAGCTTGATGGGAGTCGGTTTCGCG-3'<br>5'-CATGCATGGAATTCGAATCAAGTTTGGCGGCTC-3'	This study
pUT18-*wecA*	5'-CATGCATGAAGCTTGATGGGAGTCGGTTTCGCG-3'<br>5'-CATGCATGGAATTCGAATCAAGTTTGGCGGCTC-3'	This study
pUT18c-*wecA*	5'-CATGCATGTCTAGAGATGGGAGTCGGTTTCGCG-3'<br>5'-CATGCATGGAATTCTTAATCAAGTTTGGCGGCT-3'	This study
pKT25-*wbbL*	5'-CATGCATGCTGCAGGGGTGATCACAGTGACCTA-3'<br>5'-CATGCATGGAATTCCTAAGAGGCTTTCGTTCTC-3'	This study
pKNT25-*wbbL*	5'-CATGCATGCTGCAGGGTGATCACAGTGACCTAT-3'<br>5'-CATGCATGGAATTCGAAGAGGCTTTCGTTCTCA-3'	This study
pUT18-*wbbL*	5'- CATGCATGCTGCAGGGTGATCACAGTGACCTAT-3'<br>5'-CATGCATGGAATTCGAAGAGGCTTTCGTTCTCA-3'	This study
pUT18c-*wbbL*	5'-CATGCATGCTGCAGGGTGATCACAGTGACCTAT-3'<br>5'-CATGCATGGAATTCCTAAGAGGCTTTCGTTCTC-3'	This study
pKT25-*glfT1*	5'-CATGCATGCTGCAGGGATGGCACAAACCACTAC-3'<br>5'-CATGCATGGGTACCCTAGGGCCTATTGAATTTC-3'	This study
pKNT25-*glfT1*	5'-CATGCATGCTGCAGGATGGCACAAACCACTACC-3'<br>5'-CATGCATGGGTACCCGGGGCCTATTGAATTTCT-3'	This study
pUT18-*glfT1*	5'-CATGCATGCTGCAGGATGGCACAAACCACTACC-3'<br>5'-CATGCATGGGTACCCGGGGCCTATTGAATTTCT-3'	This study
pUT18c-*glfT1*	5'-CATGCATGCTGCAGGATGGCACAAACCACTACC-3'<br>5'-CATGCATGGGTACCCTAGGGCCTATTGAATTTC-3'	This study

pKT25-*glfT2*	5'-CATGCATGCTGCAGGGATGAAGGGTGAAGATAC-3'	This study
	5'-CATGCATGGGTACCTTATTGCTCATCGAAGACC-3'	
pKNT25-*glfT2*	5'-CATGCATGCTGCAGGATGAAGGGTGAAGATACG-3'	This study
	5'-CATGCATGGGTACCCCTTGCTCATCGAAGACCT-3'	
pUT18-*glfT2*	5'-CATGCATGCTGCAGGATGAAGGGTGAAGATACG-3'	This study
	5'-CATGCATGGGTACCCCTTGCTCATCGAAGACCT-3'	
pUT18c-*glfT2*	5'-CATGCATGCTGCAGGATGAAGGGTGAAGATACG-3'	This study
	5'-CATGCATGCTGCAGGGATGAAGGGTGAAGATAC-3'	
pKT25-*aftA*	5'-CATGCATGCTGCAGGGATGATTAACACCTCTGA-3'	This study
	5'-CATGCATGGGTACCTTACTCATTGTGCGTTACC-3'	
pKNT25-*aftA*	5'-CATGCATGCTGCAGGATGATTAACACCTCTGAA-3'	This study
	5'-CATGCATGGGTACCCCCTCATTGTGCGTTACCA-3'	
pUT18-*aftA*	5'-CATGCATGCTGCAGGATGATTAACACCTCTGAA-3'	This study
	5'-CATGCATGGGTACCCCCTCATTGTGCGTTACCA-3'	
pUT18c-*aftA*	5'-CATGCATGCTGCAGGATGATTAACACCTCTGAA-3'	This study
	5'-CATGCATGCTGCAGGGATGATTAACACCTCTGA-3'	
pKT25-*aftB*	5'-CATGCATGTCTAGAGATGACGTTTAGCCCCCAG-3'	This study
	5'-CATGCATGGAATTCTTACTGAGAGCTATATAAA-3'	
pKNT25-*aftB*	5'-CATGCATGTCTAGAGATGACGTTTAGCCCCCAG-3'	This study
	5'-CATGCATGGAATTCGACTGAGAGCTATATAAAG-3'	
pUT18-*aftB*	5'-CATGCATGTCTAGAGATGACGTTTAGCCCCCAG-3'	This study
	5'-CATGCATGGAATTCGACTGAGAGCTATATAAAG-3'	
pUT18c-*aftB*	5'-CATGCATGTCTAGAGATGACGTTTAGCCCCCAG-3'	This study
	5'-CATGCATGGAATTCTTACTGAGAGCTATATAAA-3'	
pKT25-*aftC*	5'-CATGCATGCTGCAGGGATGTTGTTGATGGCGCA-3'	This study
	5'-CATGCATGGGTACCTCATGCTGTCCTCTCAAGA-3'	
pKNT25-*aftC*	5'-CATGCATGCTGCAGGATGTTGTTGATGGCGCAT-3'	This study
	5'-CATGCATGGGTACCCGTGCTGTCCTCTCAAGAT-3'	
pUT18-*aftC*	5'-CATGCATGCTGCAGGATGTTGTTGATGGCGCAT-3'	This study
	5'-CATGCATGGGTACCCGTGCTGTCCTCTCAAGAT-3'	
pUT18c-*aftC*	5'-CATGCATGCTGCAGGATGTTGTTGATGGCGCAT-3'	This study
	5'-CATGCATGGGTACCTCATGCTGTCCTCTCAAGA-3'	
pKT25-*aftD*	5'-CATGCATGCTGCAGGGGTGCTGGGTTTTGTGGT-3'	This study
	5'-CATGCATGCCCGGGTTAGCGCTTTGGAGGCCTT-3'	
pKNT25-*aftD*	5'-CATGCATGCTGCAGGGTGCTGGGTTTTGTGGTG-3'	This study
	5'-CATGCATGCCCGGGGGCGCTTTGGAGGCCTTAA-3'	
pUT18-*aftD*	5'-CATGCATGCTGCAGGGTGCTGGGTTTTGTGGTG-3'	This study
	5'-CATGCATGCCCGGGGGCGCTTTGGAGGCCTTAA-3'	
pUT18c-*aftD*	5'-CATGCATGCTGCAGGGTGCTGGGTTTTGTGGTG-3'	This study
	5'-CATGCATGCTGCAGGGGTGCTGGGTTTTGTGGT-3'	
pKT25-*ubiA*	5'-CATGCATGTCTAGAGGTGAGCGAACACGCCGCT-3'	This study
	5'-CATGCATGGAATTCTCAAAACATCGGCATGATG-3'	
pKNT25-*ubiA*	5'-CATGCATGAAGCTTGGTGAGCGAACACGCCGCT-3'	This study
	5'-CATGCATGGAATTCGAAAACATCGGCATGATGT-3'	
pUT18-*ubiA*	5'-CATGCATGAAGCTTGGTGAGCGAACACGCCGCT-3'	This study
	5'-CATGCATGGAATTCGAAAACATCGGCATGATGT-3'	
pUT18c-*ubiA*	5'-CATGCATGTCTAGAGGTGAGCGAACACGCCGCT-3'	This study
	5'-CATGCATGGAATTCTCAAAACATCGGCATGATG-3'	
pKT25-*dprE1*	5'-CATGCATGTCTAGAGATGAACAGTTCTCACGGC-3'	This study
	5'-CATGCATGGGTACCTTAAGAAAGCTCAAGTCG-3'	

pKNT25-*dprE1*	5'-CATGCATGGCATGCCATGAACAGTTCTCACGGC-3'<br>5'-CATGCATGGGTACCCGAGAAAGCTCAAGTCGGC-3'	This study
pUT18-*dprE1*	5'-CATGCATGGCATGCCATGAACAGTTCTCACGGC-3'<br>5'-CATGCATGGGTACCCGAGAAAGCTCAAGTCGGC-3'	This study
pUT18c-*dprE1*	5'-CATGCATGTCTAGAGATGAACAGTTCTCACGGC-3'<br>5'-CATGCATGGGTACCTTAAGAAAGCTCAAGTCG-3'	This study
pKT25-*dprE2*	5'-CATGCATGTCTAGAGATGCTTAACGCAGTGGGC-3'<br>5'-CATGCATGGAATTCTTAGAACGGCAGCTTGCGG-3'	This study
pKNT25-*dprE2*	5'-CATGCATGTCTAGAGATGCTTAACGCAGTGGGC-3'<br>5'-CATGCATGGAATTCGAGAACGGCAGCTTGCGGA-3'	This study
pUT18-*dprE2*	5'-CATGCATGTCTAGAGATGCTTAACGCAGTGGGC-3'<br>5'-CATGCATGGAATTCGAGAACGGCAGCTTGCGGA-3'	This study
pUT18c-*dprE2*	5'-CATGCATGTCTAGAGATGCTTAACGCAGTGGGC-3'<br>5'-CATGCATGGAATTCTTAGAACGGCAGCTTGCGG-3'	This study
pKT25-*emb*	5'-CATGCATGTCTAGAGATGCGCCAAGTCGGTGGT-3'<br>5'-CATGCATGGAATTCTTATTCATCTACCTTCATA-3'	This study
pKNT25-*emb*	5'-CATGCATGTCTAGAGATGCGCCAAGTCGGTGGT-3'<br>5'-CATGCATGGAATTCGATTCATCTACCTTCATAT-3'	This study
pUT18-*emb*	5'-CATGCATGTCTAGAGATGCGCCAAGTCGGTGGT-3'<br>5'-CATGCATGGAATTCGATTCATCTACCTTCATAT-3'	This study
pUT18c-*emb*	5'-CATGCATGTCTAGAGATGCGCCAAGTCGGTGGT-3'<br>5'-CATGCATGGAATTCTTATTCATCTACCTTCATA-3'	This study

[a]Plasmids contain the sequence generated by PCR amplification with the pair of primer on the right column
[b]Restriction enzyme sites are underlined

Table S2 Bacterial strains and plasmids used in this study

**Plasmid or strain**	**Description or genotype**	**Origin**
E. coli XL-1 Blue	Cloning strain	Lab Stock
E. coli BTH101	F^-, *cya*-99, *ara*D139, *gal*E15, *gal*K16, *rps*L1 (Str^r), *hsd*R2, *mcr*A1, *mcr*B1 strain	[38]
pKT25	Cloning and expression vector, pSU40 derivative with T25 domain of CyaA, multiclonal sequence site (MCS) at the 3' end of T25, Kan^r	[38]
pKNT25	Cloning and expression vector, pSU40 derivative with T25 domain of CyaA, MCS at the 3' start of T25, Kan^r	[38]
pUT18	Cloning and expression vector, pUC19 derivative with T18 domain of CyaA, MCS at the 3' start of T18, Amp^r	[38]
pUT18c	Cloning and expression vector, pUC19 derivative with T18 domain of Cya, MCS at the 3' end of T18, Amp^r	[38]
pKT25-zip	Control plasmid, T25 domain of Cya fused in frame with leucine zipper of GCN4, Kan^r	[38]
pUT18c-zip	Control plasmid, T18 domain of Cya fused in frame with leucine zipper of GCN4, Amp^r	[38]
pKT25-*wecA*	pKT25 plasmid with *cyaAT25-wecA* fusion, Kan^r	This study

pKNT25-*wecA*	pKNT25 plasmid with *wecA-cyaAT25* fusion, Kan^r	This study
pUT18-*wecA*	pUT18 plasmid with *wecA-cyaAT18* fusion, Amp^r	This study
pUT18c-*wecA*	pUT18c plasmid with *cyaAT18-wecA* fusion, Amp^r	This study
pKT25-*wbbL*	pKT25 plasmid with *cyaAT25-wbbL* fusion, Kan^r	This study
pKNT25-*wbbL*	pKNT25 plasmid with *wbbL-cyaAT25* fusion, Kan^r	This study
pUT18-*wbbL*	pUT18 plasmid with *wbbL-cyaAT18* fusion, Amp^r	This study
pUT18c-*wbbL*	pUT18c plasmid with *cyaAT18-wbbL* fusion, Amp^r	This study
pKT25-*glfT1*	pKT25 plasmid with *cyaAT25-glfT1* fusion, Kan^r	This study
pKNT25-*glfT1*	pKNT25 plasmid with *glfT1-cyaAT25* fusion, Kan^r	This study
pUT18-*glfT1*	pUT18 plasmid with *glfT1-cyaAT18* fusion, Amp^r	This study
pUT18c-*glfT1*	pUT18c plasmid with *cyaAT18-glfT1* fusion, Amp^r	This study
pKT25-*glfT2*	pKT25 plasmid with *cyaAT25-glfT2* fusion, Kan^r	This study
pKNT25-*glfT2*	pKNT25 plasmid with *glfT2-cyaAT25* fusion, Kan^r	This study
pUT18-*glfT2*	pUT18 plasmid with *glfT2-cyaAT18* fusion, Amp^r	This study
pUT18c-*glfT2*	pUT18c plasmid with *cyaAT18-glfT2* fusion, Amp^r	This study
pKT25-*aftA*	pKT25 plasmid with *cyaAT25-aftA* fusion, Kan^r	This study
pKNT25-*aftA*	pKNT25 plasmid with *aftA-cyaAT25* fusion, Kan^r	This study
pUT18-*aftA*	pUT18 plasmid with *aftA-cyaAT18* fusion, Amp^r	This study
pUT18c-*aftA*	pUT18c plasmid with *cyaAT18-aftA* fusion, Amp^r	This study
pKT25-*aftB*	pKT25 plasmid with *cyaAT25-aftB* fusion, Kan^r	This study
pKNT25-*aftB*	pKNT25 plasmid with *aftB-cyaAT25* fusion, Kan^r	This study
pUT18-*aftB*	pUT18 plasmid with *aftB-cyaAT18* fusion, Amp^r	This study
pUT18c-*aftB*	pUT18c plasmid with *cyaAT18-aftB* fusion, Amp^r	This study
pKT25-*aftC*	pKT25 plasmid with *cyaAT25-aftC* fusion, Kan^r	This study
pKNT25-*aftC*	pKNT25 plasmid with *aftC-cyaAT25* fusion, Kan^r	This study
pUT18-*aftC*	pUT18 plasmid with *aftC-cyaAT18* fusion, Amp^r	This study
pUT18c-*aftC*	pUT18c plasmid with *cyaAT18-aftC* fusion, Amp^r	This study
pKT25-*aftD*	pKT25 plasmid with *cyaAT25-aftD* fusion, Kan^r	This study
pKNT25-*aftD*	pKNT25 plasmid with *aftD-cyaAT25* fusion, Kan^r	This study
pUT18-*aftD*	pUT18 plasmid with *aftD-cyaAT18* fusion, Amp^r	This study
pUT18c-*aftD*	pUT18c plasmid with *cyaAT18-aftD* fusion, Amp^r	This study
pKT25-*ubiA*	pKT25 plasmid with *cyaAT25-ubiA* fusion, Kan^r	This study
pKNT25-*ubiA*	pKNT25 plasmid with *ubiA-cyaAT25* fusion, Kan^r	This study
pUT18-*ubiA*	pUT18 plasmid with *ubiA-cyaAT18* fusion, Amp^r	This study
pUT18c-*ubiA*	pUT18c plasmid with *cyaAT18-ubiA* fusion, Amp^r	This study
pKT25-*dprE1*	pKT25 plasmid with *cyaAT25-dprE1* fusion, Kan^r	This study
pKNT25-*dprE1*	pKNT25 plasmid with *dprE1-cyaAT25* fusion, Kan^r	This study
pUT18-*dprE1*	pUT18 plasmid with *dprE1-cyaAT18* fusion, Amp^r	This study
pUT18c-*dprE1*	pUT18c plasmid with *cyaAT18-dprE1* fusion, Amp^r	This study
pKT25-*dprE2*	pKT25 plasmid with *cyaAT25-dprE2* fusion, Kan^r	This study
pKNT25-*dprE2*	pKNT25 plasmid with *dprE2-cyaAT25* fusion, Kan^r	This study
pUT18-*dprE2*	pUT18 plasmid with *dprE2-cyaAT18* fusion, Amp^r	This study
pUT18c-*dprE2*	pUT18c plasmid with *cyaAT18-dprE2* fusion, Amp^r	This study
pKT25-*emb*	pKT25 plasmid with *cyaAT25-emb* fusion, Kan^r	This study
pKNT25-*emb*	pKNT25 plasmid with *emb-cyaAT25* fusion, Kan^r	This study
pUT18-*emb*	pUT18 plasmid with *emb-cyaAT18* fusion, Amp^r	This study
pUT18c-*emb*	pUT18c plasmid with *cyaAT18-emb* fusion, Amp^r	This study

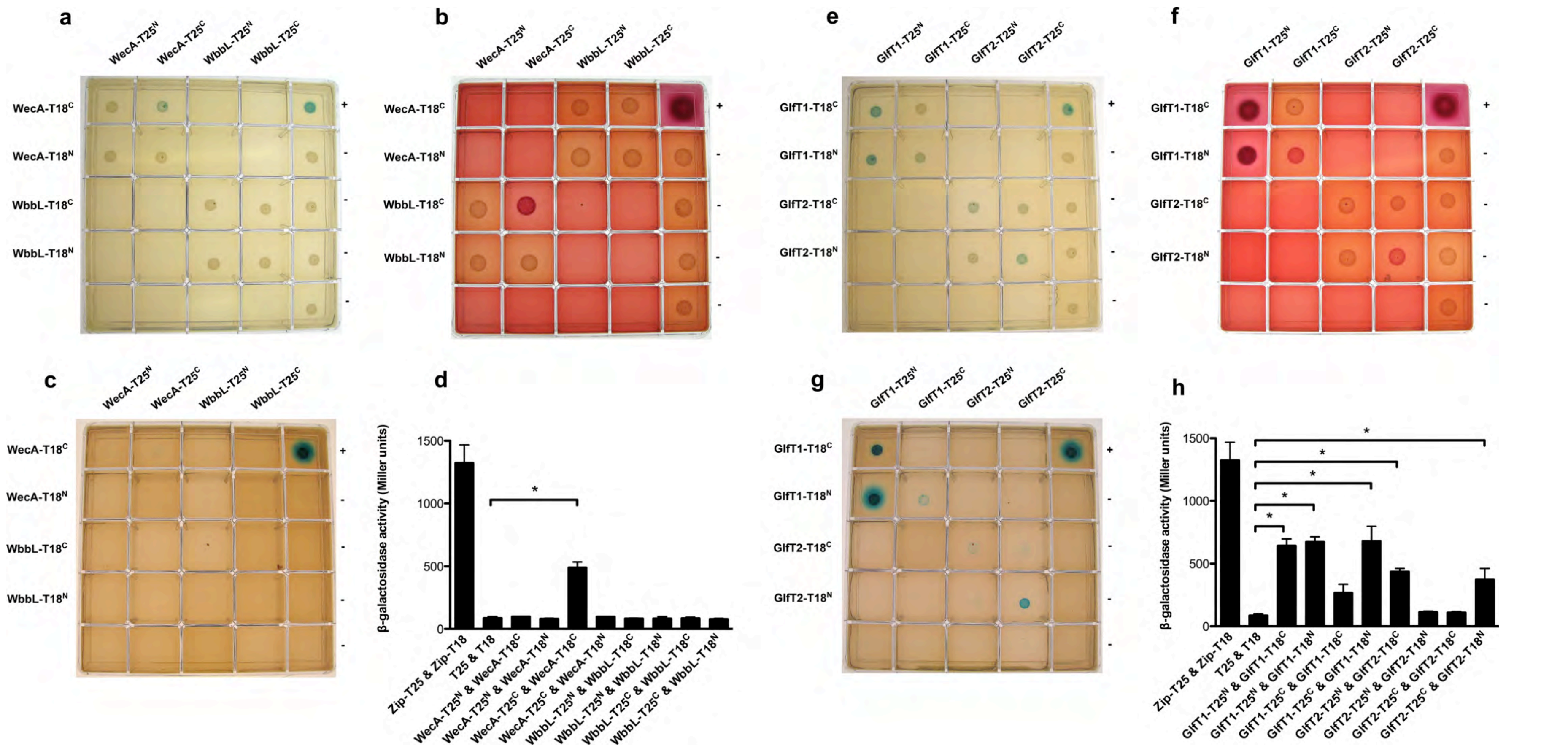

Fig. S1 BACTH analysis of self-interactions of WecA, WbbL, GlfT1, and GlfT2 from *C. glutamicum*. The genes encoding full-length proteins were fused in frame with adenylate cyclase T25 or T18 fragments at N- or C-terminus and expressed in *E. coli cya*$^{-}$ BTH101. Co-transformants containing two plasmids encoding putative interaction partners were spotted onto selective LB (**a, e**), MacConkey (**b, f**) and M63 (**c, g**) agar, as described in Materials and Methods. Protein-protein interactions are indicated by blue/red colonies through the reconstitution of adenylate cyclase catalytic domain. A strain co-expressing T25 and T18 fragments fused to leucine zipper domain was used as positive control (+), whereas empty pKT25-pUT18, pKT25-pUT18c, pKNT25-pUT18, and pKNT25-pUT18c were used as negative controls (-). **d, h** The efficiencies of functional complementation between hybrid proteins were quantified by measuring β-galactosidase activities in suspensions of toluene treated *E. coli* BTH101 harboring the corresponding plasmids. Results are expressed in Miller units and are the mean ± standard deviation of at least three independent experiments. Statistical significance was determined by Student's t-test ($p<0.01$)

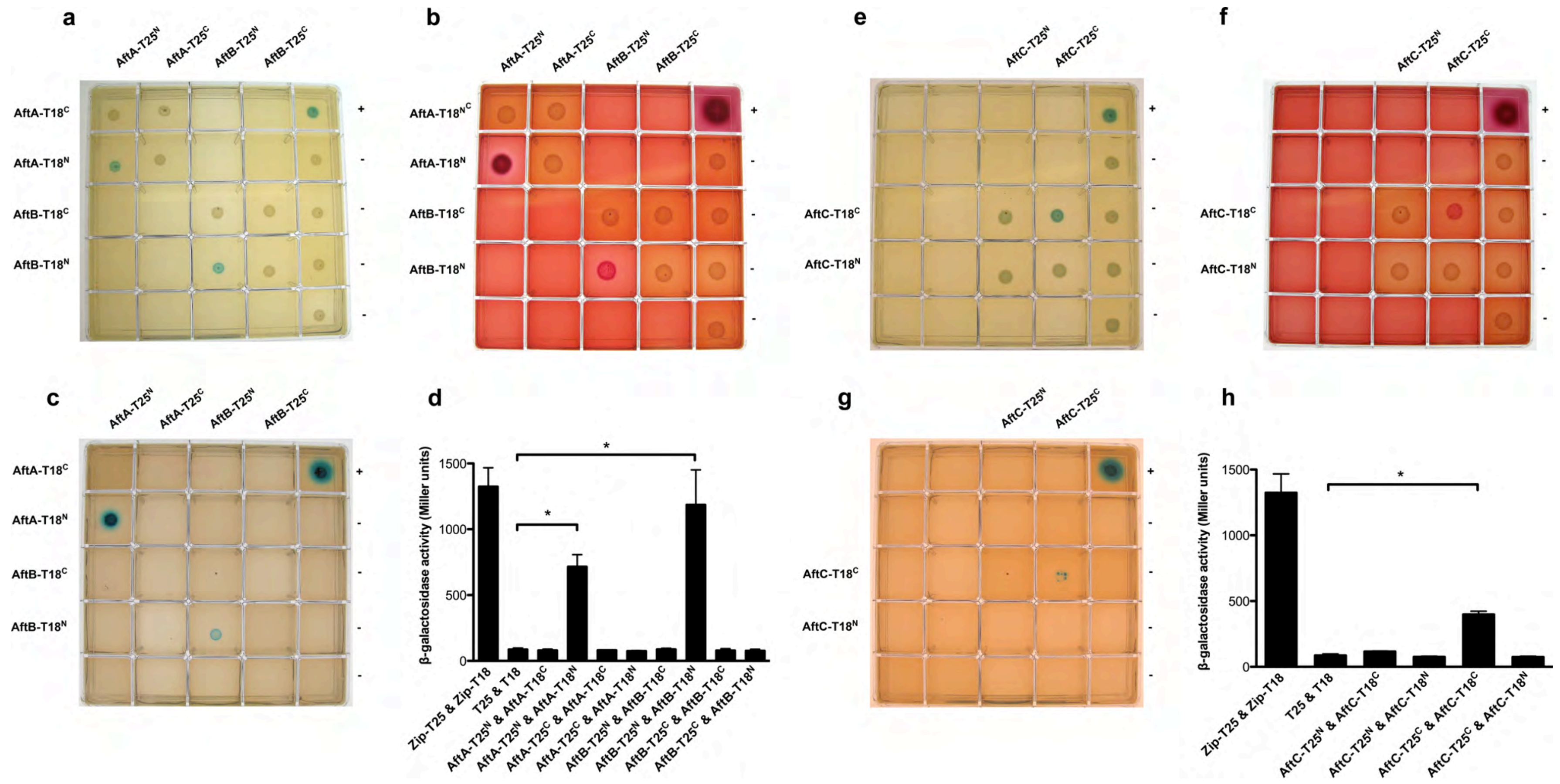

Fig. S2 BACTH analysis of self-interactions of AftA, AftB, and AftC from *C. glutamicum*. The genes encoding full-length proteins were fused in frame with adenylate cyclase T25 or T18 fragments at N- or C-terminus and expressed in *E. coli cya*$^-$ BTH101. Co-transformants containing two plasmids encoding putative interaction partners were spotted onto selective LB (**a, e**), MacConkey (**b, f**) and M63 (**c, g**) agar, as described in Materials and Methods. Protein-protein interactions are indicated by blue/red colonies through the reconstitution of adenylate cyclase catalytic domain. A strain co-expressing T25 and T18 fragments fused to leucine zipper domain was used as positive control (+), whereas empty pKT25-pUT18, pKT25-pUT18c, pKNT25-pUT18, and pKNT25-pUT18c were used as negative controls (-). **d, h** The efficiencies of functional complementation between hybrid proteins were quantified by measuring β-galactosidase activities in suspensions of toluene treated *E. coli* BTH101 harboring the corresponding plasmids. Results are expressed in Miller units and are the mean ± standard deviation of at least three independent experiments. Statistical significance was determined by Student's t-test ($p<0.01$)

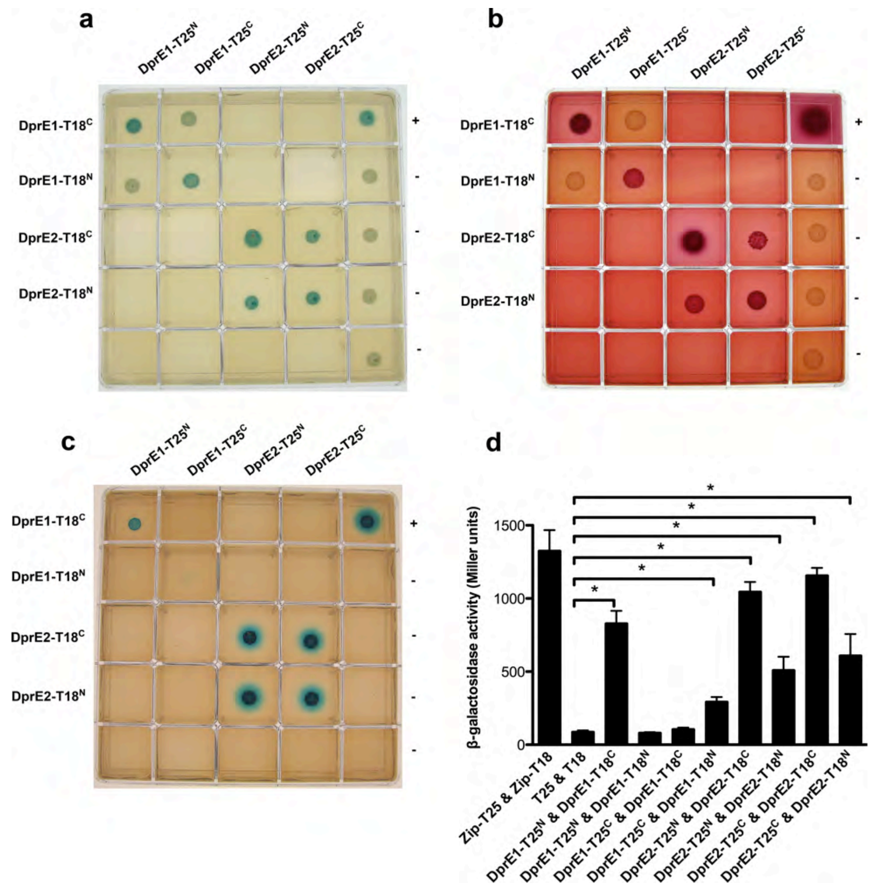

Fig. S3 BACTH analysis of self-interactions of DprE1 and DprE2 from *C. glutamicum*. The genes encoding full-length proteins were fused in frame with adenylate cyclase T25 or T18 fragments at N- or C-terminus and expressed in *E. coli cya*$^{-}$ BTH101. Co-transformants containing two plasmids encoding putative interaction partners were spotted onto selective LB (**a, e**), MacConkey (**b, f**) and M63 (**c, g**) agar, as described in Materials and Methods. Protein-protein interactions are indicated by blue/red colonies through the reconstitution of adenylate cyclase catalytic domain. A strain co-expressing T25 and T18 fragments fused to leucine zipper domain was used as positive control (+), whereas empty pKT25-pUT18, pKT25-pUT18c, pKNT25-pUT18, and pKNT25-pUT18c were used as negative controls (-). **d, h** The efficiencies of functional complementation between hybrid proteins were quantified by measuring β-galactosidase activities in suspensions of toluene treated *E. coli* BTH101 harboring the corresponding plasmids. Results are expressed in Miller units and are the mean ± standard deviation of at least three independent experiments. Statistical significance was determined by Student's t-test ($p<0.01$)

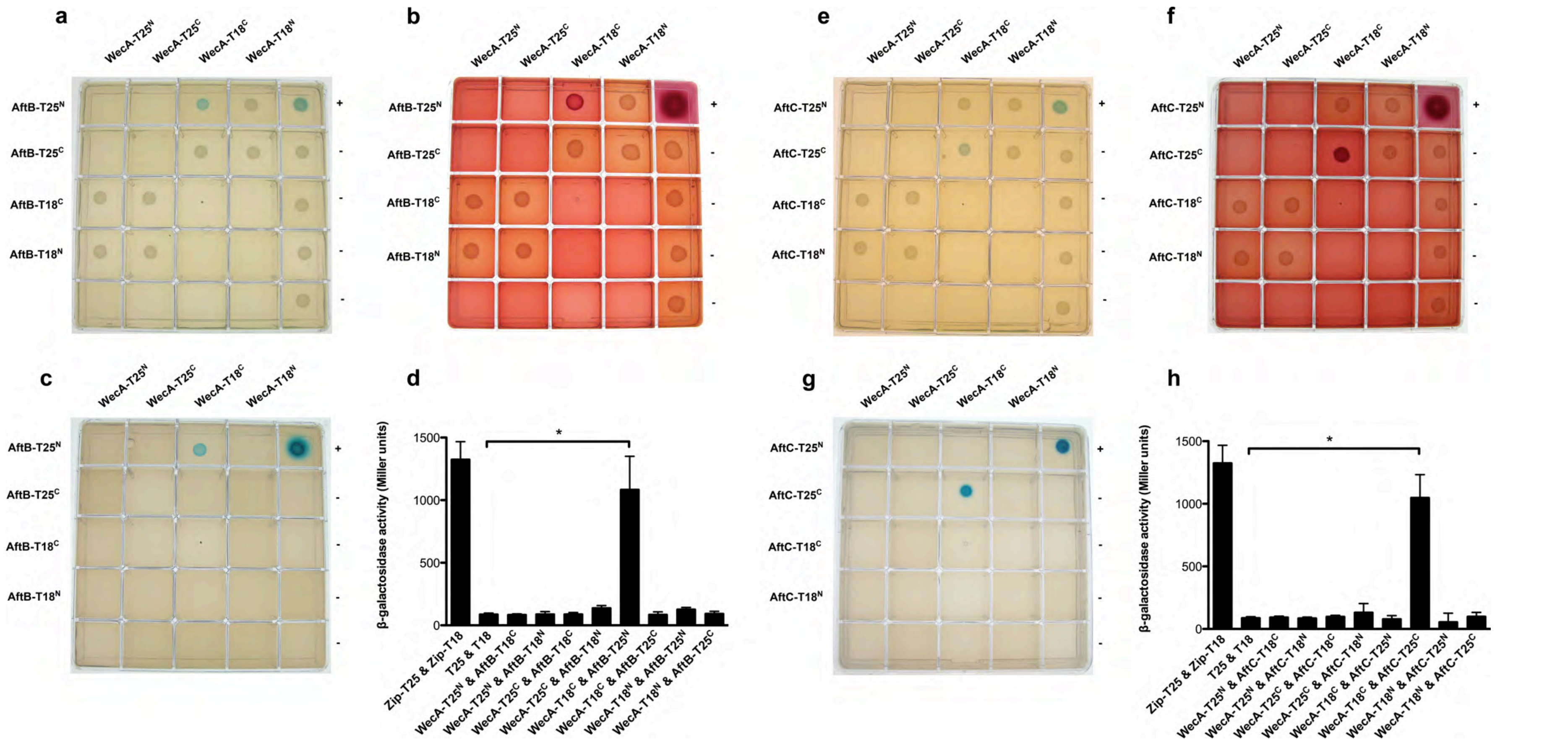

Fig. S4 BACTH analysis of interactions between WecA-AftB and WecA-AftC from *C. glutamicum*. The genes encoding full-length proteins were fused in frame with adenylate cyclase T25 or T18 fragments at N- or C-terminus and expressed in *E. coli cya*$^-$ BTH101. Co-transformants containing two plasmids encoding putative interaction partners were spotted onto selective LB (**a, e**), MacConkey (**b, f**) and M63 (**c, g**) agar, as described in Materials and Methods. Protein-protein interactions are indicated by blue/red colonies through the reconstitution of adenylate cyclase catalytic domain. A strain co-expressing T25 and T18 fragments fused to leucine zipper domain was used as positive control (+), whereas empty pKT25-pUT18, pKT25-pUT18c, pKNT25-pUT18, and pKNT25-pUT18c were used as negative controls (-). **d, h** The efficiencies of functional complementation between hybrid proteins were quantified by measuring β-galactosidase activities in suspensions of toluene treated *E. coli* BTH101 harboring the corresponding plasmids. Results are expressed in Miller units and are the mean ± standard deviation of at least three independent experiments. Statistical significance was determined by Student's t-test ($p<0.01$)

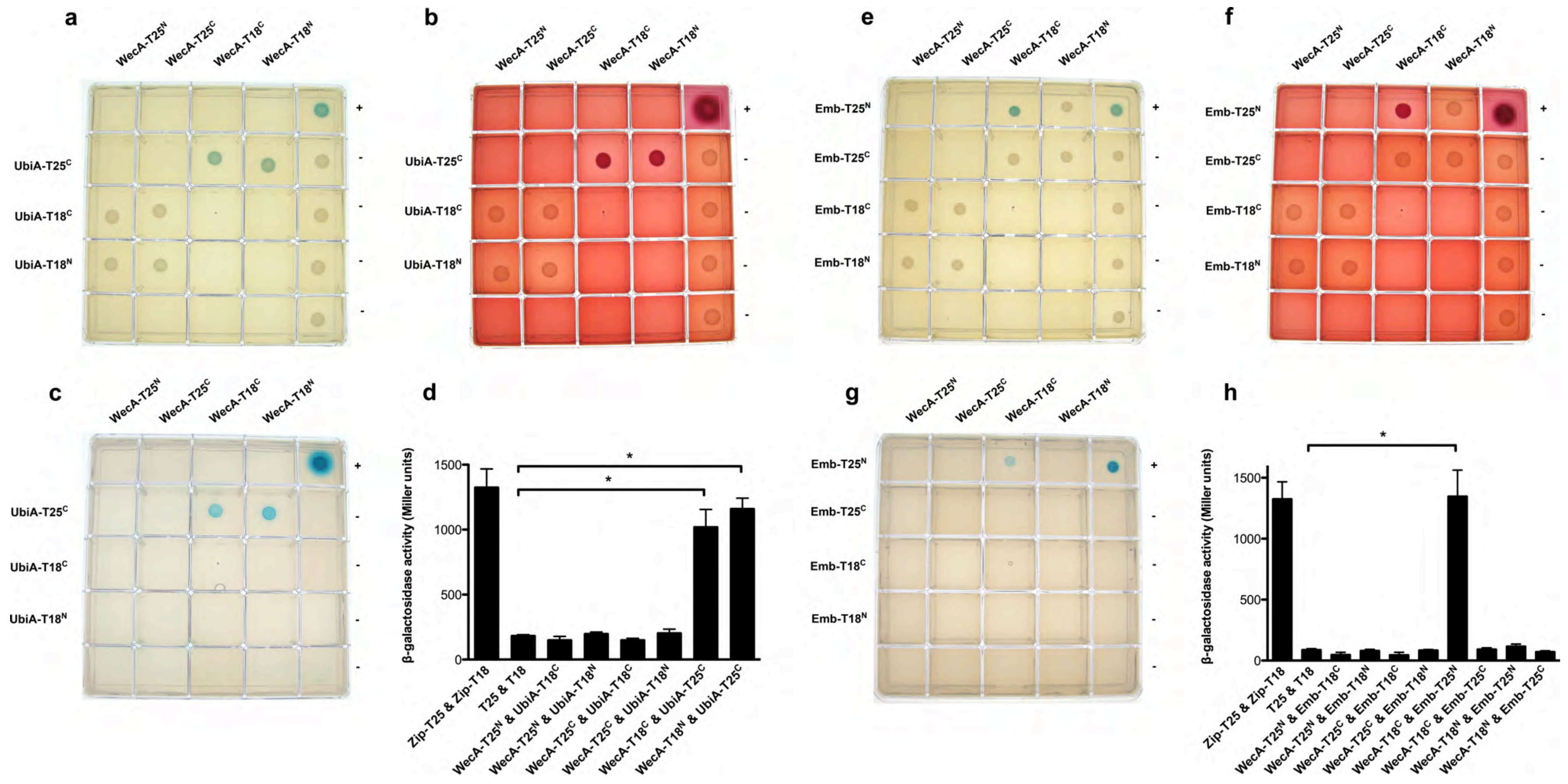

Fig. S5 BACTH analysis of interactions between WecA-UbiA and WecA-Emb from *C. glutamicum*. The genes encoding full-length proteins were fused in frame with adenylate cyclase T25 or T18 fragments at N- or C-terminus and expressed in *E. coli cya*$^-$ BTH101. Co-transformants containing two plasmids encoding putative interaction partners were spotted onto selective LB (**a, e**), MacConkey (**b, f**) and M63 (**c, g**) agar, as described in Materials and Methods. Protein-protein interactions are indicated by blue/red colonies through the reconstitution of adenylate cyclase catalytic domain. A strain co-expressing T25 and T18 fragments fused to leucine zipper domain was used as positive control (+), whereas empty pKT25-pUT18, pKT25-pUT18c, pKNT25-pUT18, and pKNT25-pUT18c were used as negative controls (-). **d, h** The efficiencies of functional complementation between hybrid proteins were quantified by measuring β-galactosidase activities in suspensions of toluene treated *E. coli* BTH101 harboring the corresponding plasmids. Results are expressed in Miller units and are the mean ± standard deviation of at least three independent experiments. Statistical significance was determined by Student's t-test ($p<0.01$)

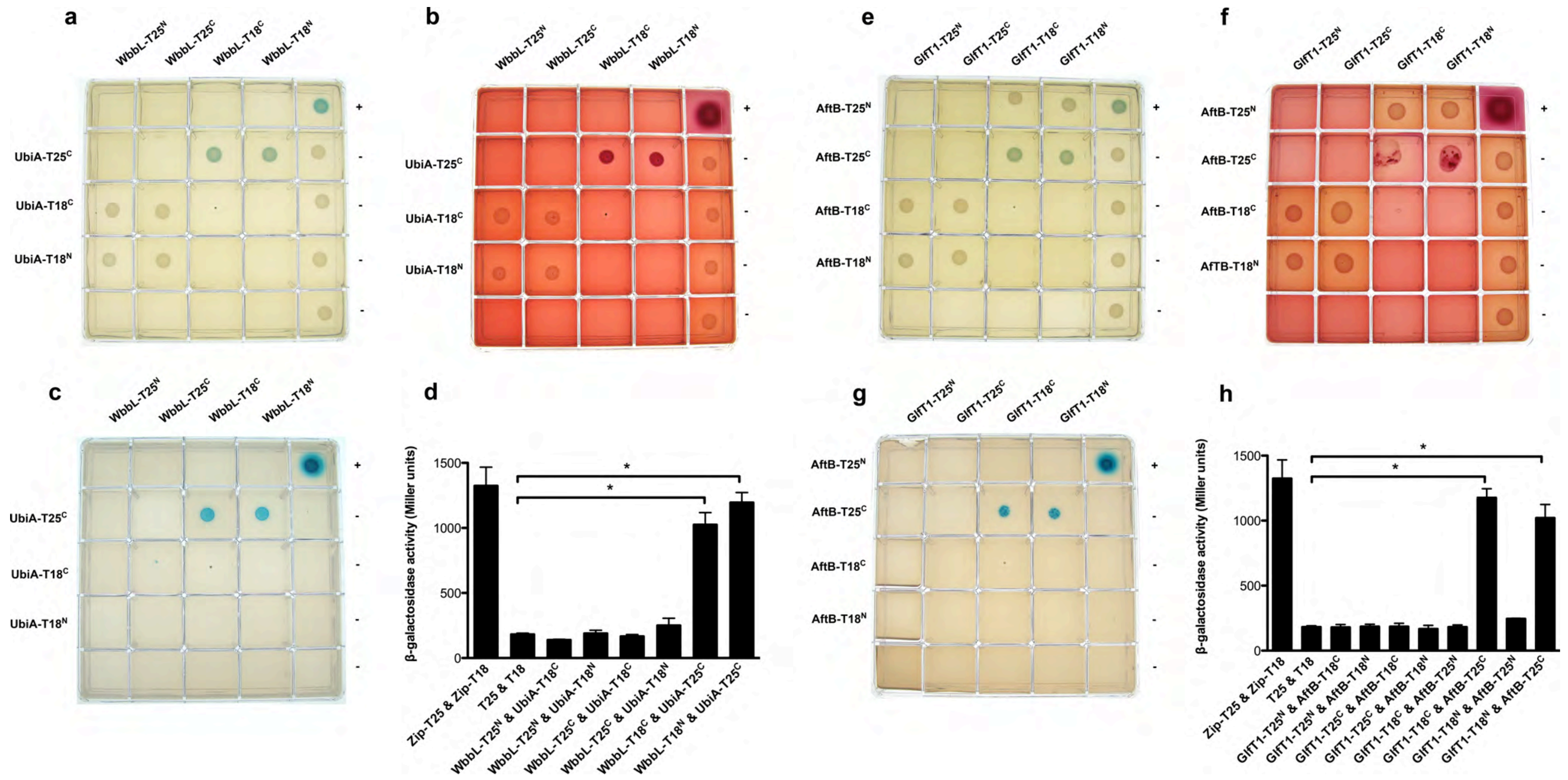

Fig. S6 BACTH analysis of interactions between WbbL-UbiA and GlfT1-AftB from *C. glutamicum*. The genes encoding full-length proteins were fused in frame with adenylate cyclase T25 or T18 fragments at N- or C-terminus and expressed in *E. coli* cya^- BTH101. Co-transformants containing two plasmids encoding putative interaction partners were spotted onto selective LB (**a, e**), MacConkey (**b, f**) and M63 (**c, g**) agar, as described in Materials and Methods. Protein-protein interactions are indicated by blue/red colonies through the reconstitution of adenylate cyclase catalytic domain. A strain co-expressing T25 and T18 fragments fused to leucine zipper domain was used as positive control (+), whereas empty pKT25-pUT18, pKT25-pUT18c, pKNT25-pUT18, and pKNT25-pUT18c were used as negative controls (-). **d, h** The efficiencies of functional complementation between hybrid proteins were quantified by measuring β-galactosidase activities in suspensions of toluene treated *E. coli* BTH101 harboring the corresponding plasmids. Results are expressed in Miller units and are the mean ± standard deviation of at least three independent experiments. Statistical significance was determined by Student's t-test ($p<0.01$)

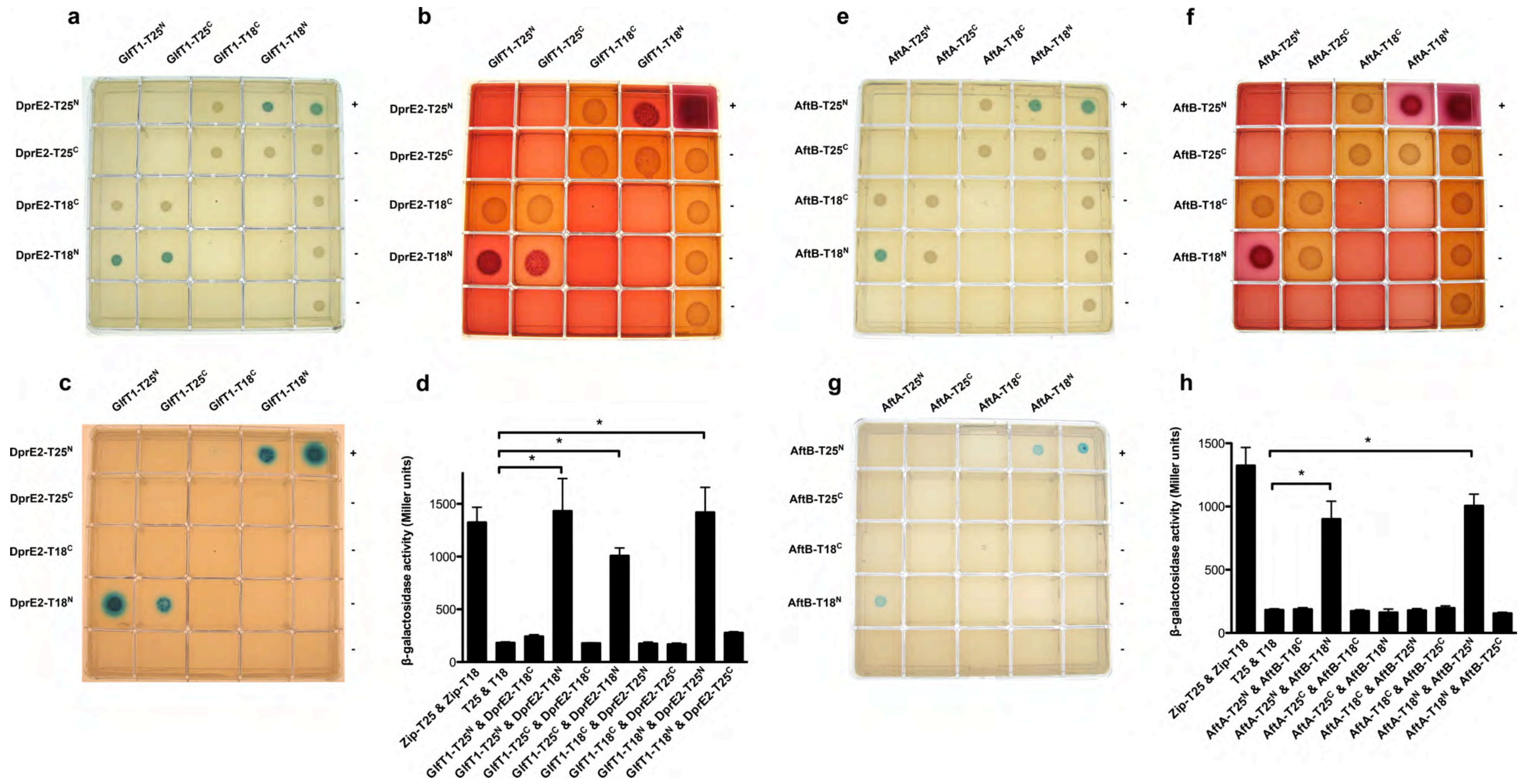

Fig. S7 BACTH analysis of interactions between GlfT1-DprE2 and AftA-AftB from *C. glutamicum*. The genes encoding full-length proteins were fused in frame with adenylate cyclase T25 or T18 fragments at N- or C-terminus and expressed in *E. coli cya⁻* BTH101. Co-transformants containing two plasmids encoding putative interaction partners were spotted onto selective LB (**a, e**), MacConkey (**b, f**) and M63 (**c, g**) agar, as described in Materials and Methods. Protein-protein interactions are indicated by blue/red colonies through the reconstitution of adenylate cyclase catalytic domain. A strain co-expressing T25 and T18 fragments fused to leucine zipper domain was used as positive control (+), whereas empty pKT25-pUT18, pKT25-pUT18c, pKNT25-pUT18, and pKNT25-pUT18c were used as negative controls (-). **d, h** The efficiencies of functional complementation between hybrid proteins were quantified by measuring β-galactosidase activities in suspensions of toluene treated *E. coli* BTH101 harboring the corresponding plasmids. Results are expressed in Miller units and are the mean ± standard deviation of at least three independent experiments. Statistical significance was determined by Student's t-test ($p<0.01$)

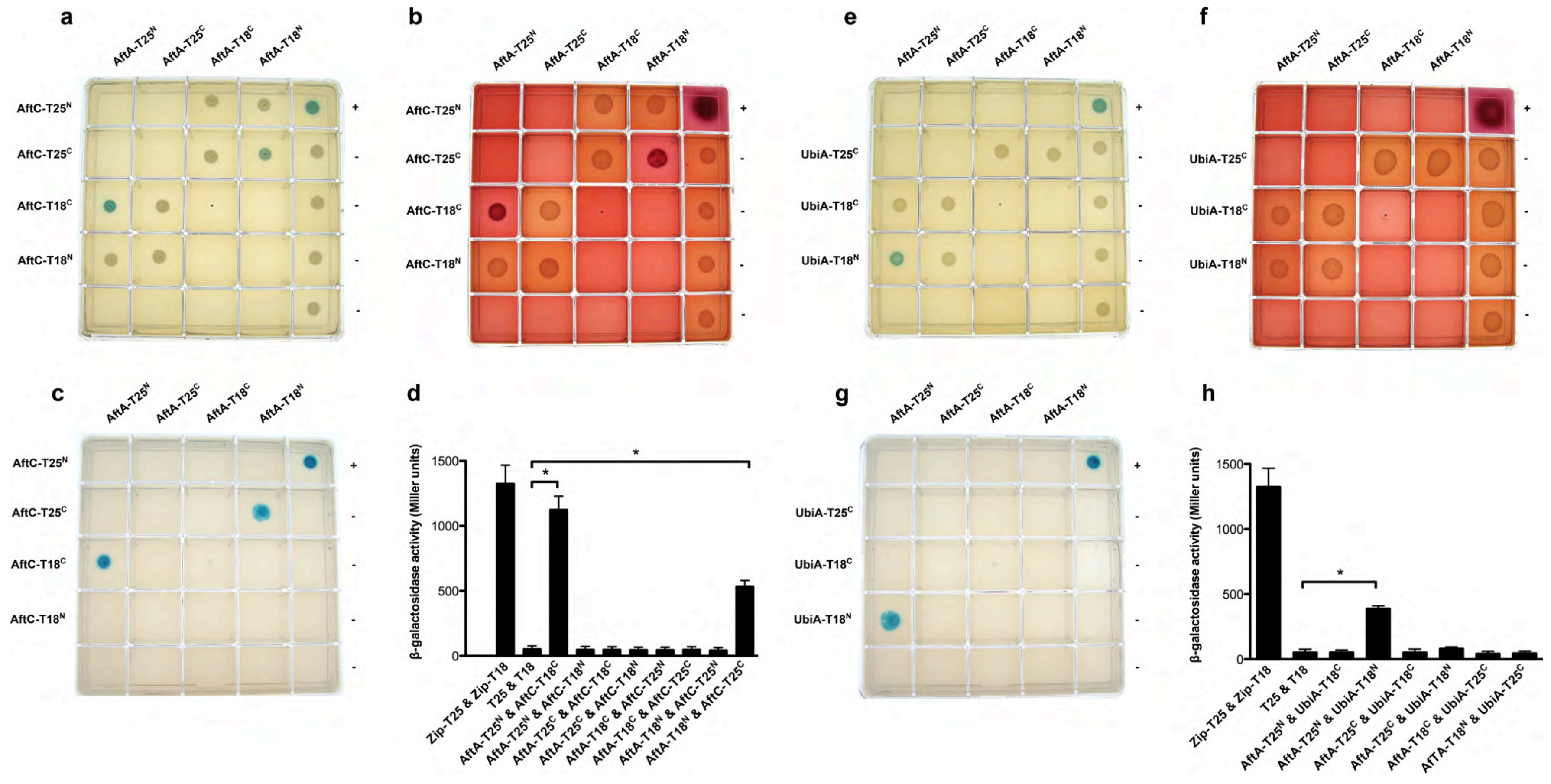

Fig. S8 BACTH analysis of interactions between AftA-AftC and AftA-UbiA from *C. glutamicum*. The genes encoding full-length proteins were fused in frame with adenylate cyclase T25 or T18 fragments at N- or C-terminus and expressed in *E. coli cya*$^-$ BTH101. Co-transformants containing two plasmids encoding putative interaction partners were spotted onto selective LB (**a, e**), MacConkey (**b, f**) and M63 (**c, g**) agar, as described in Materials and Methods. Protein-protein interactions are indicated by blue/red colonies through the reconstitution of adenylate cyclase catalytic domain. A strain co-expressing T25 and T18 fragments fused to leucine zipper domain was used as positive control (+), whereas empty pKT25-pUT18, pKT25-pUT18c, pKNT25-pUT18, and pKNT25-pUT18c were used as negative controls (-). **d, h** The efficiencies of functional complementation between hybrid proteins were quantified by measuring β-galactosidase activities in suspensions of toluene treated *E. coli* BTH101 harboring the corresponding plasmids. Results are expressed in Miller units and are the mean ± standard deviation of at least three independent experiments. Statistical significance was determined by Student's t-test ($p<0.01$)

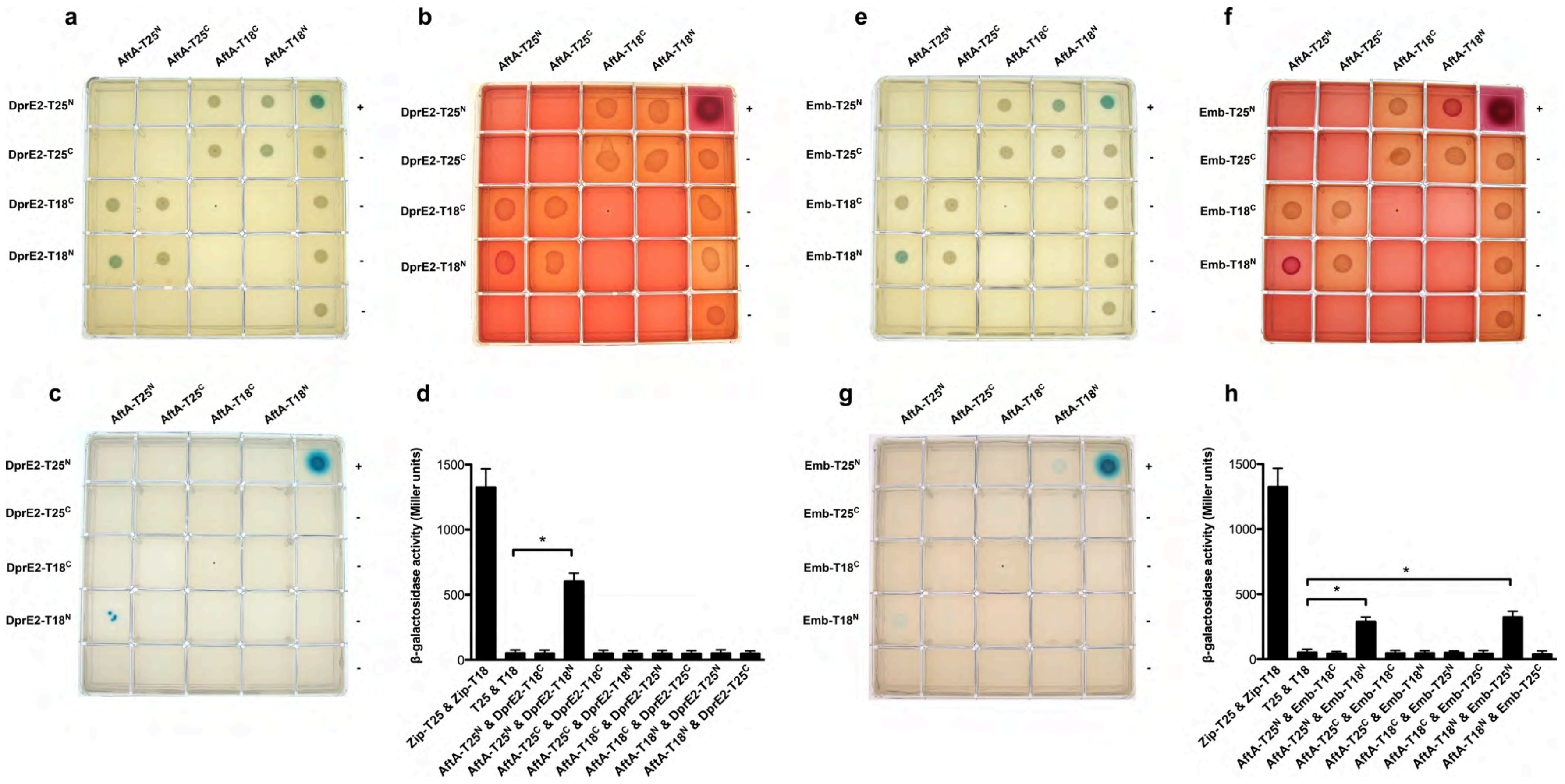

Fig. S9 BACTH analysis of interactions between AftA-DprE2 and AftA-Emb from *C. glutamicum*. The genes encoding full-length proteins were fused in frame with adenylate cyclase T25 or T18 fragments at N- or C-terminus and expressed in *E. coli* cya^- BTH101. Co-transformants containing two plasmids encoding putative interaction partners were spotted onto selective LB (**a, e**), MacConkey (**b, f**) and M63 (**c, g**) agar, as described in Materials and Methods. Protein-protein interactions are indicated by blue/red colonies through the reconstitution of adenylate cyclase catalytic domain. A strain co-expressing T25 and T18 fragments fused to leucine zipper domain was used as positive control (+), whereas empty pKT25-pUT18, pKT25-pUT18c, pKNT25-pUT18, and pKNT25-pUT18c were used as negative controls (-). **d, h** The efficiencies of functional complementation between hybrid proteins were quantified by measuring β-galactosidase activities in suspensions of toluene treated *E. coli* BTH101 harboring the corresponding plasmids. Results are expressed in Miller units and are the mean ± standard deviation of at least three independent experiments. Statistical significance was determined by Student's t-test ($p<0.01$)

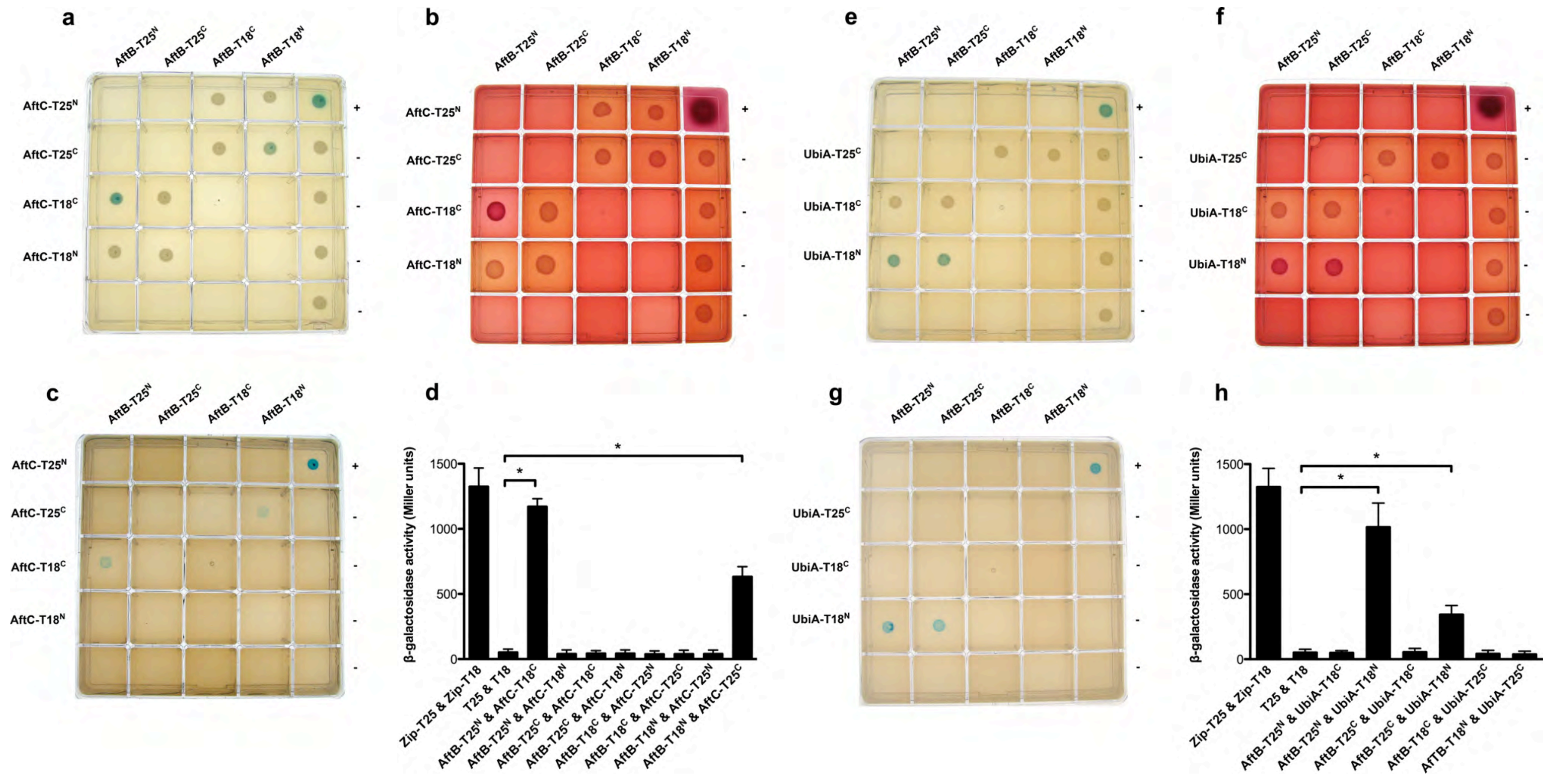

Fig. S10 BACTH analysis of interactions between AftB-AftC and AftB-UbiA from *C. glutamicum*. The genes encoding full-length proteins were fused in frame with adenylate cyclase T25 or T18 fragments at N- or C-terminus and expressed in *E. coli cya*$^-$ BTH101. Co-transformants containing two plasmids encoding putative interaction partners were spotted onto selective LB (**a, e**), MacConkey (**b, f**) and M63 (**c, g**) agar, as described in Materials and Methods. Protein-protein interactions are indicated by blue/red colonies through the reconstitution of adenylate cyclase catalytic domain. A strain co-expressing T25 and T18 fragments fused to leucine zipper domain was used as positive control (+), whereas empty pKT25-pUT18, pKT25-pUT18c, pKNT25-pUT18, and pKNT25-pUT18c were used as negative controls (-). **d, h** The efficiencies of functional complementation between hybrid proteins were quantified by measuring β-galactosidase activities in suspensions of toluene treated *E. coli* BTH101 harboring the corresponding plasmids. Results are expressed in Miller units and are the mean ± standard deviation of at least three independent experiments. Statistical significance was determined by Student's t-test ($p<0.01$)

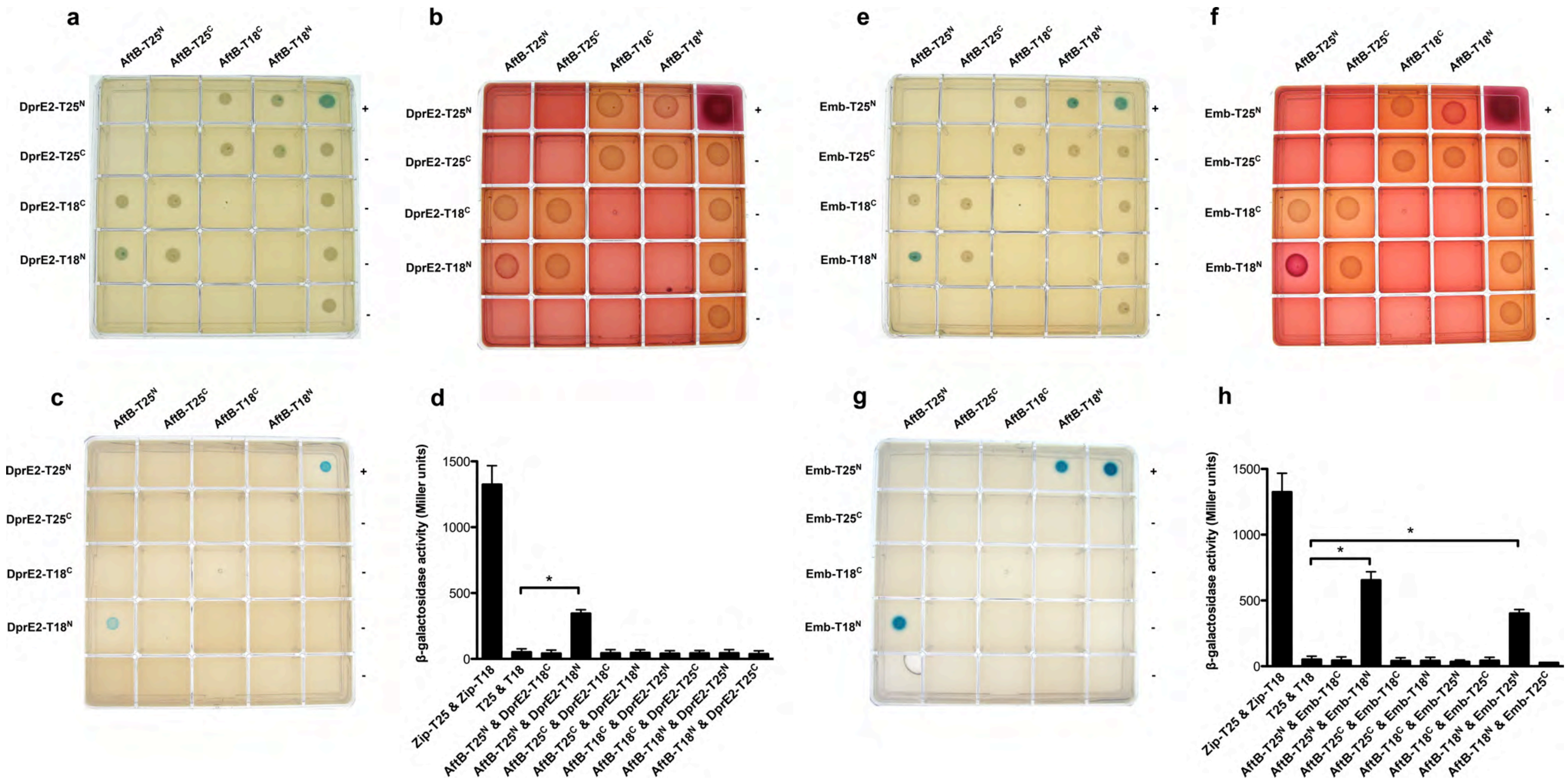

Fig. S11 BACTH analysis of interactions between AftB-DprE2 and AftB-Emb from *C. glutamicum*. The genes encoding full-length proteins were fused in frame with adenylate cyclase T25 or T18 fragments at N- or C-terminus and expressed in *E. coli cya*$^-$ BTH101. Co-transformants containing two plasmids encoding putative interaction partners were spotted onto selective LB (**a, e**), MacConkey (**b, f**) and M63 (**c, g**) agar, as described in Materials and Methods. Protein-protein interactions are indicated by blue/red colonies through the reconstitution of adenylate cyclase catalytic domain. A strain co-expressing T25 and T18 fragments fused to leucine zipper domain was used as positive control (+), whereas empty pKT25-pUT18, pKT25-pUT18c, pKNT25-pUT18, and pKNT25-pUT18c were used as negative controls (-). **d, h** The efficiencies of functional complementation between hybrid proteins were quantified by measuring β-galactosidase activities in suspensions of toluene treated *E. coli* BTH101 harboring the corresponding plasmids. Results are expressed in Miller units and are the mean ± standard deviation of at least three independent experiments. Statistical significance was determined by Student's t-test ($p<0.01$)

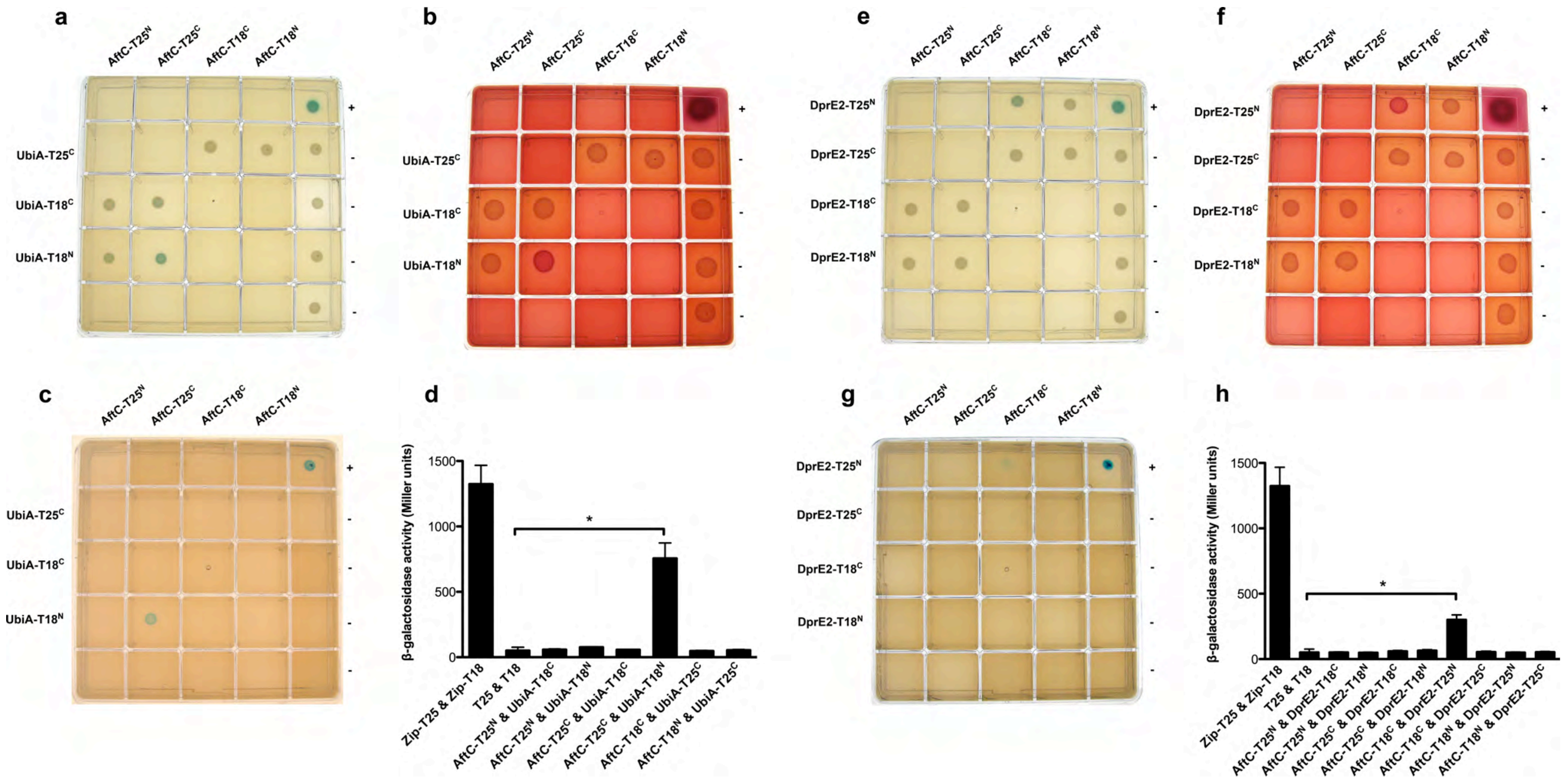

Fig. S12 BACTH analysis of interactions between AftC-UbiA and AftC-DprE2 from *C. glutamicum*. The genes encoding full-length proteins were fused in frame with adenylate cyclase T25 or T18 fragments at N- or C-terminus and expressed in *E. coli cya*$^-$ BTH101. Co-transformants containing two plasmids encoding putative interaction partners were spotted onto selective LB (**a, e**), MacConkey (**b, f**) and M63 (**c, g**) agar, as described in Materials and Methods. Protein-protein interactions are indicated by blue/red colonies through the reconstitution of adenylate cyclase catalytic domain. A strain co-expressing T25 and T18 fragments fused to leucine zipper domain was used as positive control (+), whereas empty pKT25-pUT18, pKT25-pUT18c, pKNT25-pUT18, and pKNT25-pUT18c were used as negative controls (-). **d, h** The efficiencies of functional complementation between hybrid proteins were quantified by measuring β-galactosidase activities in suspensions of toluene treated *E. coli* BTH101 harboring the corresponding plasmids. Results are expressed in Miller units and are the mean ± standard deviation of at least three independent experiments. Statistical significance was determined by Student's t-test ($p<0.01$)

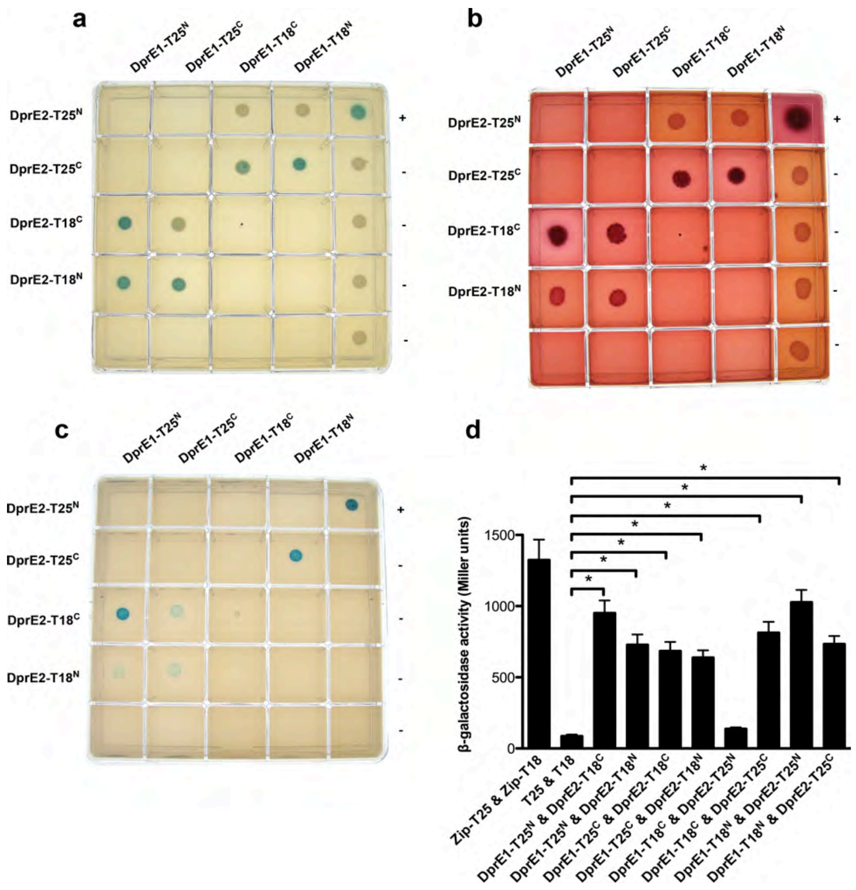

Fig. S13 BACTH analysis of interactions between DprE1-DprE2 from *C. glutamicum*. The genes encoding full-length proteins were fused in frame with adenylate cyclase T25 or T18 fragments at N- or C-terminus and expressed in *E. coli cya*$^-$ BTH101. Co-transformants containing two plasmids encoding putative interaction partners were spotted onto selective LB (**a, e**), MacConkey (**b, f**) and M63 (**c, g**) agar, as described in Materials and Methods. Protein-protein interactions are indicated by blue/red colonies through the reconstitution of adenylate cyclase catalytic domain. A strain co-expressing T25 and T18 fragments fused to leucine zipper domain was used as positive control (+), whereas empty pKT25-pUT18, pKT25-pUT18c, pKNT25-pUT18, and pKNT25-pUT18c were used as negative controls (-). **d, h** The efficiencies of functional complementation between hybrid proteins were quantified by measuring β-galactosidase activities in suspensions of toluene treated *E. coli* BTH101 harboring the corresponding plasmids. Results are expressed in Miller units and are the mean ± standard deviation of at least three independent experiments. Statistical significance was determined by Student's t-test ($p<0.01$)
